# New Strategies to Overcome Present CRISPR/Cas9 Limitations in Apple and Pear: Efficient Dechimerization and Base Editing

**DOI:** 10.3390/ijms22010319

**Published:** 2020-12-30

**Authors:** Jaiana Malabarba, Elisabeth Chevreau, Nicolas Dousset, Florian Veillet, Julie Moizan, Emilie Vergne

**Affiliations:** 1 Institut Agro, University Angers, INRAE, IRHS, SFR 4207 QuaSaV, 49000 Angers, France; jaiana.malabarba@inrae.fr (J.M.); elisabeth.chevreau@inrae.fr (E.C.); nicolas.dousset@inrae.fr (N.D.); julie.moizan@inrae.fr (J.M.); 2 IGEPP, INRAE, Institut Agro, University Rennes, 29260 Ploudaniel, France; florian.veillet@inrae.fr

**Keywords:** apple, pear, CRISPR, chimera, dechimerization, base editing, *PDS*, *ALS*

## Abstract

Despite recent progress, the application of CRISPR/Cas9 in perennial plants still has many obstacles to overcome. Our previous results with CRISPR/Cas9 in apple and pear indicated the frequent production of phenotypic and genotypic chimeras, after editing of the *phytoene desaturase* (*PDS*) gene conferring albino phenotype. Therefore, our first objective was to determine if adding an adventitious regeneration step from leaves of the primary transgenic plants (T0) would allow a reduction in chimerism. Among hundreds of adventitious buds regenerated from a variegated T0 line, 89% were homogeneous albino. Furthermore, the analysis of the target zone sequences of twelve of these regenerated lines (RT0 for “regenerated T0” lines) indicated that 99% of the RT0 alleles were predicted to produce a truncated target protein and that 67% of RT0 plants had less heterogeneous editing profiles than the T0. Base editors are CRISPR/Cas9-derived new genome-editing tools that allow precise nucleotide substitutions without double-stranded breaks. Hence, our second goal was to demonstrate the feasibility of CRISPR/Cas9 base editing in apple and pear using two easily scorable genes: *acetolactate synthase*—*ALS* (conferring resistance to chlorsulfuron) and *PDS*. The two guide RNAs under MdU3 and MdU6 promoters were coupled into a cytidine base editor harboring a cytidine deaminase fused to a nickase Cas9. Using this vector; we induced C-to-T DNA substitutions in the target genes; leading to discrete variation in the amino-acid sequence and generating new alleles. By co-editing *ALS* and *PDS* genes; we successfully obtained chlorsulfuron resistant and albino lines in pear. Overall; our work indicates that a regeneration step can efficiently reduce the initial chimerism and could be coupled with the application of base editing to create accurate genome edits in perennial plants.

## 1. Introduction

Over the last decade, gene-editing tools have emerged as new possibilities for targeted plant transformation. The most effective and least expensive method among them is the clustered regularly interspaced short palindromic repeats (CRISPR)-associated nuclease Cas9 system [1,2,3]. CRISPR/Cas9 has already accomplished a major breakthrough in animal and plant science, expanding the borders of functional gene studies and improvement of agronomical traits. Its’ triumph is based on (i) the Cas9 high rate of DNA double-strand breaks (DSBs) production, (ii) the precision of these DSBs guided by a target single-guide RNA molecule (sgRNA) creating a complex between the Cas9 nuclease and the sgRNA, and (iii) the efficiency of cell DNA repair mechanisms to create edited sequences [2,3,4]. Cas9 recognizes a protospacer adjacent motif (PAM) in the DNA sequence, mainly NGG in the case of *Streptococcus pyogenes* Cas9 (SpCas9), and destabilizes the adjacent double-stranded DNA. This step allows base pairing between 17 and 21 bp target-dependent sequence from the sgRNA and its matching target DNA sequence, followed by DSBs [5,6]. In plants the majority of DSBs are repaired by the error-prone non-homologous end-joining (NHEJ) mechanism, which creates small insertions or deletions (Indel) in the targeted sequence, potentially resulting in frameshift mutations that may trigger the nonsense-mediated decay pathway that leads to mRNA degradation. These truncated and/or non-functional proteins (knock-out) are an essential tool for gene function analysis [7,8]. CRISPR/Cas knock-out targeted mutagenesis has already been implemented in fruit tree species among different plant families: Rosaceae: apple [9], pear [10], strawberry [11], Rutaceae: sweet orange [12], Duncan grapefruit [12], Vitaceae: grape [13], Actinidiaceae: kiwifruit [14], Malvaceae: cacao [15], Musaceae: banana [16]. Furthermore, CRISPR-Cas9-mediated knock-out already led to the improvement of major traits in fruit species: fungal resistance [14,15,17], bacterial resistance [18,19], early flowering [10,20], and dwarfing [21].

Recently, it has been brought to light that even if CRISPR/Cas9 is a powerful and precise method to induce targeted mutagenesis, it also creates phenotypic and genotypic chimeric mutated plants [10,22,23]. Chimeras, also called ‘sports’ in horticulture, are organisms that possess phenotypically and/or genotypically distinct cells/tissues [24]. As with many fruit species, the transformation of apple and pear adult varieties is mainly achieved by *Agrobacterium*-mediated transformation of leaf explants followed by adventitious bud regeneration [25,26]. In this system, the regenerated transgenic plants do not arise from single cells, which can lead to the formation of chimera of T-DNA integration events [27] and the frequency of these chimeric transgenic lines can be high; e.g., 12% in Citrus [28]. When genetic transformation is used to stably express a CRISPR/Cas9 editing system, Cas9 is active during the whole length of the organogenesis process (up to 6 months in apple and pear) and this creates a mosaic of different edition events between cell lineages composing each transgenic regenerated line. In apple, this type of genotypic chimera occurs at high frequency (>85%) [9,10]. The chimerism can easily be excluded by sexual propagation of the transgenic plants due to Mendelian segregation in the following generation [29]. Unfortunately, this step cannot be applied to highly heterozygous, self-incompatible, and vegetatively propagated species, such as apple and pear [30,31]. Therefore, alternative strategies for chimerism elimination must be developed. Adventitious shoot regeneration has already been applied to segregate natural chimeras in pear [32,33] and could offer a functional alternative to reduce editing chimerism in apple and pear.

Although CRISPR/Cas9 is a feasible and straightforward technique to transform eukaryotic species by gene knock-out, the introduction of point mutations for gain of function required template DNA and homology-directed repair (HDR), until recently. Unfortunately, precise HDR is very inefficient in plants, and an accurate single-base changes or substitutions (base editing) system was needed [34]. Recently, CRISPR-Cas9-based genome editing systems, called base editing (BE), have been established, centered on the use of a nickase Cas9 (nCas9), or a catalytically inactive Cas9 endonuclease (dead Cas9, dCas9), fused to an adenine deaminase (ABE) or a cytidine deaminase (CBE), creating precise base substitutions of A-to-G or C-to-T on the non-complementary strand, respectively, and devoid of DSBs [35,36,37,38]. In the first step, cytosine or adenine is converted to uracil or inosine, respectively. Later, during DNA replication uracil and inosine is then converted to thymine and guanine, respectively, even though different conversions have also been reported, e.g., C-to-G and C-to-A [39,40,41,42,43]. To increase outcome predictability, albeit at the cost of reduced sequence diversification, a uracil DNA glycosylase inhibitor protein (UGI) can be added to the construct to prevent uracil excision and downstream error-prone repair systems, thereby increasing the ratio of C-to-T conversions and reducing the rate of Indel formation [39]. Base editors have been successful in several model and crop plant species e.g., Arabidopsis, rice, tomato, wheat, maize, oilseed rape, potato, and watermelon [41,42,43,44,45,46,47].

Apple (*Malus x domestica* Bork.) and pear (*Pyrus communis* L. and *P. pyrifolia* Nak) are among the most produced temperate fruits in the world, reaching 86 million and 23 million tons in 2018, respectively (FAOSTAT http://faostat.fao.org) and their genetic engineering could accelerate the improvement of elite varieties since both are also amenable to genetic transformation by *Agrobacterium tumefaciens*. There is a need for improvement of CRISPR/Cas9 techniques, and to our knowledge, no report of base editing via CRISPR/Cas9 has been published so far on these species. To achieve this goal, the use of scorable genes can help improve plant transformation techniques. One of them is the *acetolactate synthase* (*ALS*), which encodes an enzyme that catalyzes the initial step of the biosynthetic pathway for branched-chain amino acids, thus leucine, isoleucine, and valine [48]. Herbicides from the sulfonylurea class (e.g., chlorsulfuron, chlorimuron-ethyl, cloransulam-methyl) are ALS inhibitors, which affect the plant development causing their death [49,50]. Previous studies showed that point mutations on the *ALS* gene (e.g., P197 on *A. thaliana*, P186 on *S. lycopersicum*, D376 on *Lolium perenne* and P185 on *M. domestica*) can confer dominant resistance to ALS-inhibitors [51,52,53,54]. On that account, *ALS* can be an appropriate cis-marker gene for transgenic plant selection. Another gene is *Phytoene desaturase* (*PDS*) which is responsible for chlorophyll, carotenoid, and gibberellin biosynthesis, therefore its inactivation results in albino and dwarf phenotypes [55]. Being visually accessible makes *PDS* an attractive reporter gene for testing genome editing methods. *MdPDS* was first knocked-out by CRISPR/Cas9 technique in apple rootstock ‘JM2’ with an editing rate of 31.8% [9] and later *MdPDS* and *PcTFL1* were efficiently edited (>85% in apple) with an improved protocol on ‘Gala’ and ‘Conference’, respectively [10].

In the present work, we tackled the chimerism limitation of organogenesis-based CRISPR/Cas9 technique, proving that an adventitious regeneration step can efficiently reduce chimerism in perennials. Furthermore, we used CRISPR/Cas9 CBE to target two scorable genes in apple and pear using stable transformation. We made use of CBE for the following purposes: (i) to create a stop-codon on *PDS* resulting in a loss-of-function of the *phytoene desaturase* gene, (ii) to create an amino-acid substitution on *ALS* that results in the non-binding of the herbicide chlorsulfuron. We identified precise base conversion and amino acid substitution in *ALS* and *PDS* genes in apple and pear, resulting in plants that are chlorsulfuron resistant and albino.

## 2. Results

### 2.1. Phenotypic Dechimerization after Adventitious Regeneration of Buds from a Variegated Transgenic Line

**Figure 1:**
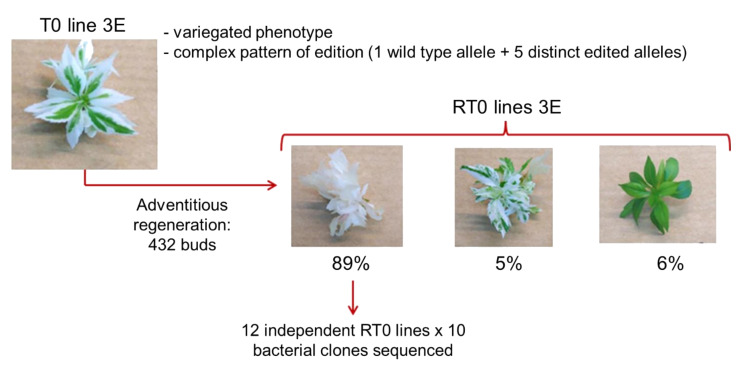
Scheme of assessment of adventitious regeneration efficiency to reduce phenotypic and editing chimerism in a CRISPR-Cas9 edited apple line.

A previous study of our group employed efficiently CRISPR/Cas9 KO of the *PDS* gene in apple by using two different sgRNAs, with the production of transgenic plants presenting three early-stage phenotypes: green (26%), albino (10%), and variegated (64%) [10]. One year of micropropagation and recurrent selection of white sectors was necessary to recover 84% of the transgenic lines presenting a pure albino phenotype. In addition to this phenotypic chimerism, sequence analysis of the transgenic albino lines revealed a high rate (88%) of *MdPDS* editing chimerism [10]. In the present study, one of our goals was to evaluate if an additional adventitious regeneration step could efficiently reduce the phenotypic and editing degree of chimerism in apple CRISPR/Cas9 transgenic plants. For this purpose, one of the T0 variegated apple mutated plants (line 3E) showing a complex pattern of *MdPDS* editing was selected to undergo an additional in vitro regeneration step (Figure 1). Twenty-four 3E leaves were wounded by scalpel and incubated on regeneration medium for organogenesis of adventitious buds. After 3 months on regeneration medium, 432 well-developed buds were phenotyped. Among them, 89% (384) were albino, 6.25% (27) were green and 4.75% (21) still presented a variegated phenotype. This indicates a very high rate of phenotypic chimerism elimination after adventitious regeneration. Among the albino regenerated plants (RT0 for “regenerated T0” lines), 12 lines were randomly selected and named 3E1 to 3E12 (Figure 1). DNA samples from T0 and RT0 lines were extracted for evaluation of *PDS* edited sequences.

### 2.2. Evidence of Edit Dechimerization in RT0 Lines

**Figure 2:**
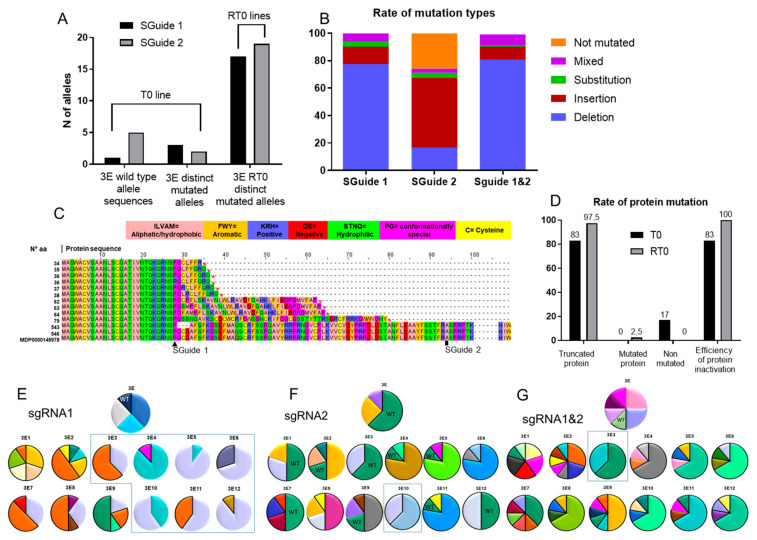
Evaluation of mutations on *PDS* gene on T0 and RT0 plants. (**A**) total number of wild type (non-mutated) and of distinct mutated alleles recovered from the 8 bacterial clones sequenced per line T0, and number of distinct (new and previously found in T0 line) mutated alleles found in the 12 RT0 lines, between single guide RNA 1 and 2; (**B**) types of mutations created with each sgRNA; (**C**) alignment of PDS protein translation by allele size on RT0 and T0 plants along with the amount of alleles found within each size; (**D**) rate of protein mutation showing the percentage of truncated protein, mutated and non-mutated, and efficiency of protein inactivation; (**E**–**G**) alleles diversity in each line represented by percentage of allele presence, wild type allele presence on T0 and RT0 lines is indicated (WT) and black borders represent the alleles supposedly derived from the WT allele; alleles present on the T0 line conserved the same color when appearing on the RT0 lines; the blue boxes indicate the biallelic RT0 lines (**E**) diversity of PDS alleles per T0 (3E) and RT0 (3E1–3E12) lines at target sgRNA1 region; (**F**) diversity of PDS alleles per T0 and RT0 lines at target sgRNA2 region; (**G**) diversity of PDS alleles per T0 and RT0 lines considering mutations on both sgRNA1 and sgRNA2 regions as one allele.

For genotypic chimerism evaluation, an initial amplification of the *PDS* gene sequence flanking the two zones targeted by the sgRNA1 and sgRNA2 [10] was performed on the DNA of T0 (3E) and RT0 (3E1 to 3E12) plants. The position of the sgRNAs on the *PDS* gene is illustrated in Figure S1. To distinguish the diversity of edits that could be present on each line, a cloning step was added for allele-specific characterization and 10 bacterial clones were sequenced per line. We observed that the sgRNA1 was more efficient than sgRNA2 for *MdPDS* editing, as observed previously [10]. Indeed, in the T0 line, when sequencing the target 1 region seven edited alleles were initially found, wherein some of these alleles shared an identical sequence and so three distinct alleles are overall recovered and counted, and one wild–type allele; while the target 2 presented only three edited alleles (two distinct) and still five wild-type sequences (Table 1 and Figure 2A). When both targets were taken into account, the T0 line presented a complex pattern of editing composed of five distinct edited alleles and one wild-type allele (Table 1). The edited alleles presented various types of edits: for example, one presented a deletion as well as a substitution, one presented a substitution and one had two substitutions as well as a deletion.

The sequencing of the RT0 lines showed an increased allelic diversity at both targets, which can be explained by new editing from the wild alleles of the T0 line and by the discovery of edits that were not initially detected in the T0 line. When taking both single guides region into account as one allele, a total of 39 distinct alleles were detected in the RT0 lines. Again, the sgRNA1 mutated almost 100% of the sequences analyzed, but the target 2 still presented 25% of not mutated sequences (Figure 2B). Among the edits of the RT0 lines, deletion (around 77% for sgRNA1 and 16% for sgRNA2) and insertions (around 12% for sgRNA1 and 50% for sgRNA2) were more frequent, followed by substitution (4.2% for sgRNA1 and 4.0% for sgRNA2) and mixed alleles (5.8% for sgRNA1 and 3.0% for sgRNA2), which contain two or more different modifications, e.g., an insertion and a substitution (Figure 2B). Nevertheless, when analyzing both guides we found that 99% of the edited alleles produced a predicted truncated PDS protein (Figure 2C,D). The whole prediction of the produced PDS proteins for the distinct edited alleles is shown in File S1. The degree of chimerism reduction was different in the two target zones. At target 1, a majority of biallelic lines (7/12) were recovered (Figure 2E) while at target 2 a majority of chimeric lines (10/12) (Figure 2F) was still observed (Table 1). The overall results considering both target zones (Figure 2G) indicated that the addition of an adventitious regeneration step allowed the elimination of wild allele and the recovery of one biallelic RT0 line (Table 1).

### 2.3. CRISPR/nCas9 Cytidine Deaminase Target Design

**Figure 3:**
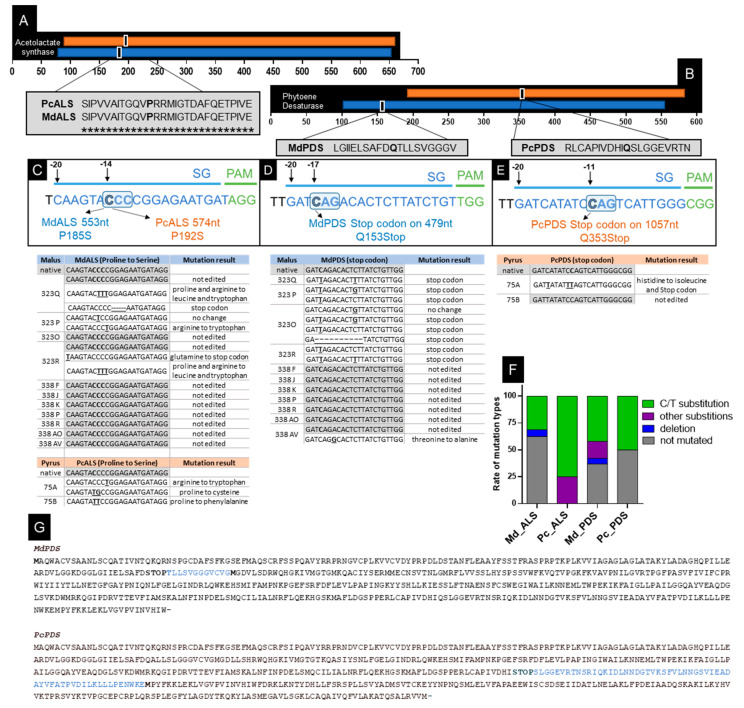
Cytidine deaminase base editing (CBE) of *ALS* and *PDS* genes in *M. domestica* and *P. communis.* (**A**) Position of each sgRNA ALS on gene (black) and on the acetolactate synthase domain for pear (orange) and apple (blue); (**B**) position of each sgRNA PDS on gene (black) and on the phytoene desaturase domain for pear (orange) and apple (blue); (**C**) single guide RNA for *ALS* gene in apple and pear; (**D**) single guide RNA for *PDS* gene in pear; (**E**) single guide RNAs for PDS gene in apple; Sanger sequencing results for the recovered lines are below C, D, E; (**F**) Rate of mutation type per sg_RNA; (**G**) MdPDS protein sequence prediction after stop codon creating by CBE, for apple and pear, marked in blue are the amino acids that are lost by the non-sense mutation and in bold is the M (methionine) showing the possible new start codon.

We first produced a CBE construct suitable for *Agrobacterium*-mediated transformation harboring nCas9_PmCDA1_UGI fusion sequence under the control of the PcUbi4-2 promoter (File S2). Apple and pear whole-genome sequences were recently made available, helping CRISPR/Cas single guides’ RNA design and selection [56,57,58,59,60]. We used the following strategy for CBE sgRNA design targeting *ALS* and *PDS* genes in *M. domestica* and *P. communis* in order to: (i) create a base editing at a specific proline position on *ALS* aiming to suppress the site for chlorsulfuron binding and *ALS* inhibition and (ii) create a stop codon on *PDS* for enzyme inactivation (thus change a **C**AA/**C**AG/**C**GA to a **T**AA/**T**AG/**T**GA). Furthermore, our goal was to co-edit both genes using a single final vector, thus in a single transformation step. The specific proline codon of *MdALS* and *PcALS*, P185 and P192 respectively, contain a CCC sequence, so the sgRNA_ALS was designed to target the first cytosine base of the proline codon (-14nt from the PAM NGG sequence; Figure 3A,B) and consequently change it to serine (**C**CC to **T**CC). The same guide was used for both species due to sequence similarity and because no off-target was detected with 0, 1, or 2 mismatches. The target nucleotide for apple sgRNA_ALS is at position 553nt—P185S and the pear is at 574nt—P192S, from the beginning of the gene (Figure 3C). For sgRNA_MdPDS a stop codon was envisioned at position 479nt—Q153stop with the change of the first cytosine base (-11nt from PAM) from the glutamine codon (**C**AG to **T**AG) (Figure 3D). For sgRNA_PcPDS the same change was performed but the sequence of the guide differs, therefore its position is located further on the gene, 1057nt—Q353stop, with the target cytosine base at -17nt from PAM sequence (Figure 3E). For both sgRNA_PDS no off-target was detected with 0, 1, 2 or 3 mismatches. Both loci of interest were sequenced in ‘Gala’ and ‘Conference’ in vitro cultures for specificity sequence confirmation. No mutations were detected in their sequences when compared to the genomic sequences available. All single guides presented a predicted specific score between 89–98 and a predicted efficiency between 50–72 [61].

### 2.4. Efficient Base Editing of Scorable Genes in Apple and Pear

By *Agrobacterium*-mediated stable transformation we obtained 16 transgenic lines for apple and 2 lines for pear with kanamycin selection medium (Table 2). From the 16 apple lines obtained, we continued analysis on 11 lines, due to their sufficient development and growth which secure further genotypic and phenotypic analysis. The 11 apple lines and the 2 pear lines were analyzed by PCR and *EF1α* gene was used as a marker of plant DNA suitability for PCR. We also used as controls the purified plasmid of each transformation (pDenMdALSPDS or pDenPcALSPDS), extracted from *A. tumefaciens* strain, and ‘Gala’ and ‘Conference’ genomic DNAs from non-transformed plants. The evaluation of *A. tumefaciens* presence on the transgenic lines was tested with UF/B1R primers which proved that all lines were free from *Agrobacterium*. The presence of the Cas9 coding sequence was also confirmed in all lines. Moreover, even if all lines survived in a kanamycin propagation medium, they were tested by PCR for the presence of the selectable marker *nptII*, and all were confirmed positive.

By Sanger sequencing of bacterial clones containing alleles of the areas targeted by the guides after mass cloning, we were able to describe the precise allele modification created by each construct in the two pear lines and in the 11 apple lines recovered. Base editing of the *ALS* gene was achieved in both species. For apple, we obtained the modification of P185 into leucine (CCC to CTT) by the editing of bases C_-13-12_-to-T (lines 323Q and 323R in Figure 3C). For pear, we observed the mutation of P192 to cysteine, P192C, therefore CCC to TGC (at position C_-14_-to-T and C_-13_-to-G) for line 75A. For the line 75B, we observed the mutation of P192 to phenylalanine, P192F, therefore CCC to TTC (at position C_-14-13_-to-T) (Figure 3C). Moreover, an adjacent modification of C_-11_-to-T also modified arginine to tryptophan (R186W) on three apple lines (323Q, 323P, and 323R). Concerning the *PDS*-targeted site, in which we aimed to create a stop codon, Sanger sequencing revealed that the majority of apple edited lines presented a C to T change at C-_17_ creating the expected stop codon (Figure 3D). Only one allele of apple line 338 AV presented a base editing (A to G) that created a change of amino acid, threonine to alanine, instead of the expected non-sense mutation (Figure 3D). For the two pear lines obtained, the *PDS* gene was efficiently base edited in one of the lines (75A), where we observed the modification of the glutamine353 for the desired stop codon (CAG to TAG at position C_-11_-to-T, therefore Q353Stop) (Figure 3E). Furthermore, in this line, we observed the base editing from C to T at position -_12_ and -_17_ from the PAM. The C_-12_-to-T consisted of a silent mutation and the C_-17_-to-T consisted of a missense non-conservative mutation that changed histidine to isoleucine (H351I). A summary of the editing profiles is given in Figure 3F and Table 3. Figure 3G shows the complete protein sequence of PDS in apple and pear, with the expected cytidine deaminase mutation for the creation of the stop codon, and the potential new reading frame (start codon).

**Figure 4:**
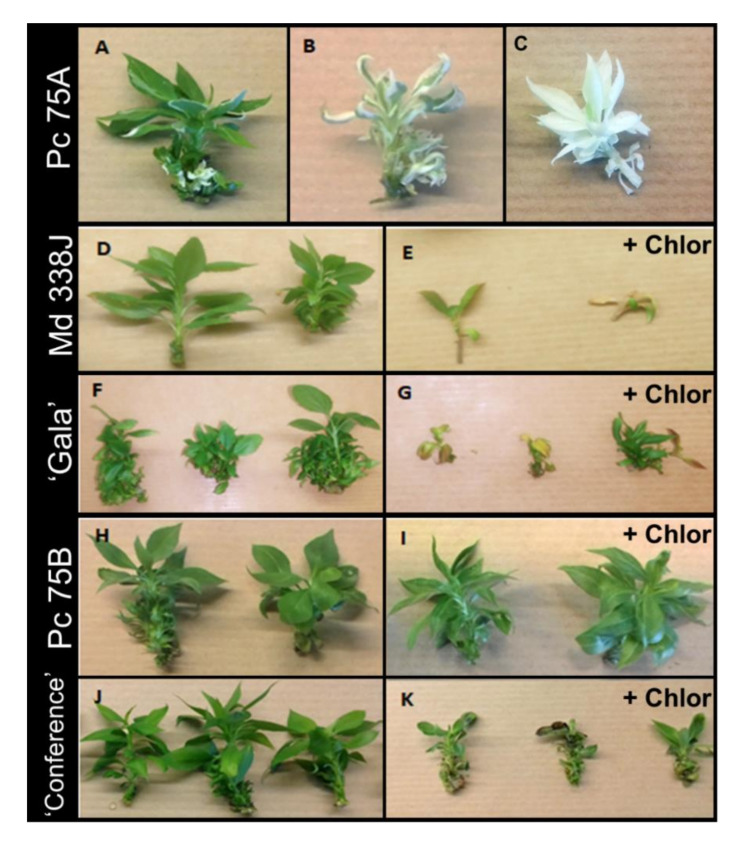
Transgenic lines phenotype for base-editing of *PDS* and *ALS* genes. PDS-related phenotypes for *P. communis* line 75A phenotype (**A**–**C**); ALS-related phenotype for *M. domestica* for line 338J (**D**,**E**) and ‘Gala’ (**F**,**G**) as control (+Chlor = 2 months on chlorsulfuron at 1 µg/L). ALS-related phenotype for *P. communis* line 75B (**H**,**I**) and ‘Conference’ as control (**J**,**K**) (+Chlor = 2 months on chlorsulfuron at 2 µg/L). *P. communis* (Pc); *M. domestica* (Md); Chlorsulfuron treatment (+Chlor).

The phenotype of the transgenic lines was assessed over a period of six to ten months after the end of all transformation experiments. The two pear lines had normal growth and morphology. One of them (75A) rapidly produced white sectors (Figure 4A), which developed after several subcultures and led to completely albino shoots. (Figure 4B,C). No white sectors appeared on the second pear line. Most apple transgenic lines had reduced growth and thinner leaves compared to the control, but no white sectors could be detected in these lines. In the absence of apple or pear chlorsulfuron-resistant reference lines, we first evaluated the susceptibility of these two species to a range of chlorsulfuron doses. Regeneration of adventitious buds from leaves of ‘Gala’ and ‘Conference’ was totally inhibited by chlorsulfuron 0.5 µg/L (Figure S3). This indicates a very high susceptibility of apple and pear to this herbicide. Among apple transgenic lines, only two lines (323Q and 338J) grew enough to permit their chlorsulfuron resistance evaluation. Both lines proved to be susceptible to chlorsulfuron 1 µg/L (Figure 4D,E) similarly to the control ‘Gala’ (Figure 4F–G). On the contrary, both pear transgenic lines (75A and 75B) proved resistant to chlorsulfuron up to 2 µg/L (Figure 4H,I), contrary to the control ‘Conference’ (Figure 4J,K).

## 3. Discussion

### 3.1. Effective Chimerism Reduction by Adventitious Regeneration

During genetic transformation by organogenesis, chimerism frequently occurs within the regenerated plants. In the case of apple and pear, this chimerism cannot be eliminated by subsequent selfing due to gametophytic self-incompatibility controlled by the single S-locus, [31,62]. Furthermore, both species present a long reproductive cycle and are highly heterozygous. Thus, their production is based on clonal propagation by grafting. The occurrence of chimerism after genetic transformation of apple and pear is further complicated by the progressive activity of Cas9 during the long process of regeneration of the edited transgenic lines. Here we applied an adventitious regeneration step to the leaves of a *PDS* CRISPR/Cas9 edited variegated line to observe if the regenerated lines would present a less chimeric pattern. The adventitious regeneration produced a majority of buds of the homogeneous phenotype (89% albino, 6.25% green) with only 4.75% of still heterogenous variegated phenotype. This indicates that this method is effective in dissociating this type of phenotypic chimera. A histological study has shown that these type of buds have their origin from new meristems formed from a few cells within the explant, probably from a single histological layer, mostly L1 or L2 [63]. Chimera dissociation tests have already been carried out on pear to separate phenotypic chimeras (plants variegated green/albino or red/green), comparing the effects of adventitious regeneration versus rapid multiplication. After adventitious regeneration, 100% of the plants tested were no longer chimeric versus only 1/3 in the case of rapid multiplication [33].

Besides, our analysis concerned the study of total or partial disappearance of edition chimeras or the appearance of new editions. Genotypic chimerism is regularly observed in studies of other fruit species. Indeed, grapevine targeted mutagenesis via CRISPR/Cas9 resulted in 100% of chimeric plants [13]. The same is true for bananas, where chimeric plants were obtained by CRISPR/Cas9 targeted mutagenesis [16]. In our work we observed new editing profiles on RT0 lines compared to the T0 line that can have different and complementary explanations: the initial data on the T0 plant was not exhaustive, and other editing profiles could have been present without being detected. This could explain the presence in a high percentage of a deletion mutation (CCTGGAT(G)CGATGCCTTTTCTTT) in RT0 lines (23%) that was not found in the T0 line. Such a percentage indicates that this mutation was probably initially present in the T0 line. Another explanation is that the T0 line was not completely edited for the *PDS* gene, still presenting a wild-type allele. Given that the gene coding for Cas9 is constitutively expressed and under the control of a CaMV35S promoter, the enzyme continues to be produced in the plant [20]. And along with the expression of the sgRNAs and the presence of a target area in the genome, the Cas9 continues to perform its endonuclease activity as long as there are unedited cells in a transgenic line. As a result, the creation of new editing profiles can still take place and be observed in RT0 lines. The wild-type alleles represent 13% of the T0 alleles (sgRNA1), but it is no longer found in RT0 lines. On the other hand, a high number of newly edited alleles was identified in the RT0 lines. In particular, the low efficiency of the sgRNA2 (71% of wild-type alleles at target 2 of the T0 line) can explain the creation of new edited alleles by the sgRNA/Cas9 complex in RT0 lines. The presence of new editing profiles has also been observed in strawberry plants when a plant edited by CRISPR/Cas9 was subjected to self-fertilization. Of the thirteen seeds obtained, ten had new mutations, proving that Cas9 is transmitted to descendants [11]. Given the lower possibility of creation of new edited alleles at target 1 (targeted by sgRNA1; only 1 wild-type allele found remaining in T0 line against 5 at target 2; Table 1), the analysis of the outcome of the zone between the T0 3E plant and the RT0 3E1 to 3E12 plants seems the best region to look at to evaluate if an additional regeneration step is effective to reduce editing chimerism. Indeed, at target 1, T0 line presents a chimerism profile, with a mix of three distinct edited alleles and the wild type one, while regenerant lines (RT0) present a majority of biallelic profiles (7/12). Thus, an additional regeneration step seems effective to reduce editing chimerism in the absence of a large amount of remaining wild type alleles. Taken together, our results indicate that applying an additional adventitious regeneration step to apple edited lines is a successful method to eliminate phenotypic chimerism and to decrease editing chimerism. A similar approach was used in poplar, where the reduction in chimeric mutants produced by CRISPR/Cas9 was obtained after a second round of shoot regeneration [64].

### 3.2. Precise Base Editing Efficiently Applied in Apple and Pear In Vitro Cultures

CRISPR/Cas9 was the major breakthrough technique for gene studies in the last decade, being implemented in numerous types of organisms such as bacteria, yeast, plants, and animals, and winning the Nobel Prize for Chemistry in 2020. In plants, this technique proved to be a great tool for efficient knock-out of a specific gene but correction of a specific gene by knock-in remained at a very low level of efficiency [34]. Therefore, Cas9 modifications creating catalytically impaired Cas9 variants plus the coupling of deaminase enzymes were envisioned for specific nucleotide sequence modification [40,65]. To demonstrate the efficiency of base editing in apple and pear, we used a CBE system, which includes a UGI sequence after the nCas9-PmCDA1 fusion, ensuring the inhibition of base excision repair and improving the base-editing outcome purity. By targeting two scorable genes at once, each of them with one single guide RNA target sequence, we observed the editing of nucleotides on the genes *ALS* and *PDS* of apple and pear.

Apple transformation methods make use of selection markers commonly derived from bacteria (e.g., *neomycin phosphotransferase II—nptII* for kanamycin resistance; *phosphinothricin acetyltransferase—bar* for glyphosinate resistance, or *phosphomannose isomerase- manA* for positive mannose selection) [66,67,68]. As a result, the regenerated plants are considered transgenic which increases public concerns towards genetically modified food particularly in the European Union [69,70]. To lighten this public concern, endogenous selection genes can be used for plant transformation, as a consequence creating cisgenics or intragenics [71,72]. Here we used the *ALS* gene as an alternative for perennial gene function studies with cis-gene as markers. In our study, we successfully edited different C-bases in the target zone of *ALS*. For pear *ALS* gene, the base editing of proline192 to cysteine (P192C-CCC to TGC) on line 75A and proline to phenylalanine (P192F-CCC to TTC) on line 75B were efficiently in conferring resistance to chlorsulfuron. These ALS proline mutations were not yet described to achieve the expected phenotype. For the apple *ALS* gene, a previous study showed that the change of proline185 (P185) conferred herbicide resistance when it was converted to alanine, serine, or histidine, but not when the P185 was deleted [54]. This substitution acceptation was also shown for different plant species such as *Schoenoplectus juncoides, Lindernia micrantha,* and *Papaver rhoeas* [73,74,75]. In our study, for the apple *ALS* gene, we obtained the substitution of proline185 to leucine (P185L-CCC to CTT), but this change in amino acid does not seem to confer chlorsulfuron resistance. The edited apple lines presented a chimerism in edits, with still non-edited alleles (or silent edits), but also alleles that were edited elsewhere than on the expected base, creating unexpected mutations. Furthermore, the phenotype of the apple transgenic lines was affected, since their growth was reduced and their leaves presented a thinner phenotype. This could indicate that the unexpected mutations created in some apple lines (P185L, R186W, and G183stop) could have affected the biosynthesis of the amino acids leucine, isoleucine, and valine by a mutated acetolactate synthase protein. Moreover, it is clear that our large range of P185 modification occurred due to cytidine deaminase activity that also changed other C bases inside the target sequence (C at positions_-13_ and _-12_ from the PAM in the sgRNA_ALS). Therefore, we suggest that a minimum of Cs should be present on a target sequence for more accurate editing. In our case, there was no option for choosing the sgRNA_ALS, given the position of the target proline and the requirement of the PAM sequence (NGG) on the BE system, which has a small base-editing window [76,77]. One possibility to overcome this difficulty is to use orthologues of the SpCas9 nickase from bacterial and archaeal species, or to use engineered variants of the SpnCas9, that present a PAM NG recognition site [37,78,79]. The use of CBEs with narrowed editing windows has also been recently developed to limit the risk of bystander mutations, increasing the precision of CBE-mediated base substitutions [80,81]. Another possibility to overcome a particular C-rich sequence is to apply an ABE to target A sites of the *ALS* gene-specific region for chlorsulfuron binding [41,42]. Previous studies have reported that CBE derived from the rat APOBEC1 deaminase can induce substantial genome-wide sgRNA-Cas9-independent off-target editing in rice [82]. To minimize these undesired effects, engineered CBEs derived from different deaminase domains have been recently developed [83], constituting interesting tools for applications of base editing in plants. Concerning the base editing of the *PDS* gene aiming for a stop codon, and therefore the production of albino plants, we obtained the expected mutations with the creation of stop codons in both species, but the expected phenotype only for pear. Surprisingly, even with the expected mutations in five apple lines we still did not observe albino or variegated leaves after one year of in vitro culture of these lines. We hypothesize that a new reading frame start site located soon after the created stop codon (12 aa) restart PDS translation in apple, creating a new protein translated without frameshift and that might be able to function alone or along with the truncated initial protein. On the contrary, the editing of pear *PDS* gene to create a stop codon has an available new reading frame after a significantly larger distance (58 aa), maybe in that case the second possible protein lacks essential amino acids and consequently, this could explain why the mutated *PcPDS* promoted the observed albino phenotype in a gradual and steady manner on the edited pear line 75A. These hypothetical proteins translated from the *PDS* mutated genes in apple and pear are illustrated in Figure S2. Possible frameshifts alike were already observed in HAP1 cells, in which, after stop codon creation by CRISPR/Cas9 generated knock-out lines, the residual protein expression was still found for about one-third of the quantified targets [84]. Furthermore, it is possible that these PDS protein modifications in apple lines explain altogether the reduced growth and thinner leaves phenotypes observed in the corresponding lines, or along with the unexpected mutated ALS proteins.

## 4. Conclusions

Our study focused on the chimeric status of CRISPR/Cas9 genetically edited trees species, an issue which cannot be resolved by sexual propagation. We concluded that an additional step of in vitro adventitious regenerations can efficiently reduce phenotypic chimerism and hence homogenize plants edition profiles, as long as the remaining wild type alleles are present in a low amount. This is an important requirement for the application of gene-editing methods to gene function studies in apple and pear, and also for functional studies with other species that are amenable to *Agrobacterium*-mediated transformation through organogenesis. In addition, we showed the efficiency of a co-base editing via CRISPR/nCas9 fused to cytidine deaminase on two scorable genes of apple and pear. Both species belong to one of the major family of fruits consumed every day worldwide. Our experiments showed that the desired mutations were introduced with the use of one single guide RNA for each gene in the same construction. The edits on the acetolactate synthase (*ALS*) gene in pear created amino-acid substitution of a specific proline and consequently, by destabilizing the herbicide binding site, conferred resistance to the herbicide chlorsulfuron. Furthermore, by editing *PDS* and *ALS* simultaneously, we proved the feasibility of targeting multiple genes with base editing method in apple and pear. Moreover, co-editing *ALS* opens a new possibility of cisgenesis, using this endogenous gene as a selection marker. To our knowledge, these are the first reports of base editing in apple and pear. Overall, our results contribute to improve genetic engineering methods for these perennial species for gene function studies as well as for future application in breeding programs.

## 5. Material and Methods

### 5.1. Biological Material

The plant material used in our study were in vitro proliferating shoot cultures of the apple ‘Gala’ and the pear ‘Conference’. ‘Gala’ plants were micropropagated on Murashige and Skoog, 1962 (MS) [85] medium supplemented with 0.5 mg/L 6-benzyladenine (BA) and 0.1 mg/L 3-indolebutyric acid (IBA), while ‘Conference’ plants were micropropagated as described by Leblay et al., 1991 [86] on a derivative of Lepoivre’s medium supplemented with 0.5 mg/L BA and 0.1 mg/L IBA. Both cultures were transferred to fresh medium every 4 weeks. All plants were cultivated in a growth chamber at 22–24 °C with a 16:8 h light:dark photoperiod (cool white fluorescent tubes, 40–60 mmol m^−2^ s^−1^).

Dechimerization assay was performed on CRISPR-Cas9-edited apple lines [10] in which the binary vector CRISPR-PDS created from the pDE-CAS9 vector [87] was used to knock-out the *Phytoene Desaturase* gene of *Malus domestica* (MD04G0021400). The knock-out construction comprised the Cas9 gene from *Streptococcus pyogenes* driven by PcUbi4-2 promoter from parsley (*Petroselinum crispum*) and terminated by the Pea3a terminator from *Pisum sativum,* followed by two gRNAs, driven by MdU3 and MdU6 promoters from *M. domestica* and terminated by a polyT terminator. The vector also contained the selection gene *nptII* controlled by *nos* promoter and terminator.

For the creation of constructs and vector cloning, One-Shot R TOP10 Chemically Competent *Escherichia coli* (Thermo Fisher Scientific, Waltham, MA, USA) were used. Plant transformation was performed with *Agrobacterium tumefaciens* strain EHA105 [88] containing the binary vector of interest along with a ternary plasmid for the expression of a constitutive *virG* gene (pBBR1MCS-5; [89]).

### 5.2. Construction of Vectors

**Figure 5:**
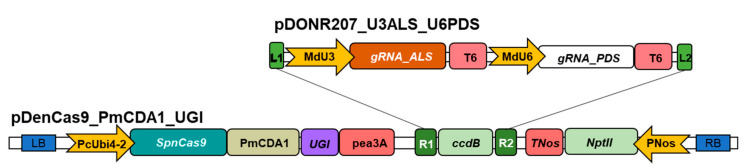
CRISPR/nCas cytidine base editing vector construction. Two single guides RNAs, specifically chosen for targeting the genes *ALS* and *PDS* were assembled on the pDORN207 vector and later introduced into final vector pDenCas9_PmCDA1_UGI by Gateway LR recombination. The nickase *Cas9* gene (*SpnCas9*) from *Streptococcus pyogenes* is driven by PcUbi4-2 promoter (P) from parsley (*Petroselinum crispum*) followed by *Petromyzon marinus* cytidine deaminase (PmCDA1) and uracil DNA glycosylase inhibitor protein (UGI) with transcription termination by the pea3A terminator (T) from pea (*Pisum sativum*). gRNA_ALS and gRNA_PDS are, respectively, driven by MdU3 and MdU6 promoters from *M. domestica* and transcription is terminated by a polyT terminator. Transformants are selected with a *nptII* gene controlled by nos promoter and terminator from *Agrobacterium tumefaciens*. L1 and L2, R1 and R2: Att sites for Gateway LR recombination. LB and RB: T-DNA borders.

The CBE binary vector used for base editing of the genes *Acetolactate synthase* (*MdALS*: MD06G1028700; *PcALS*: PCP000109) and *Phytoene desaturase* (*MdPDS*: MD04G0021400; *PcPDS*: PCP034470) in apple ‘Gala’ and pear ‘Conference’ was constructed as follow. First, the nCas9 sequence was generated from the pDeCas9 [87] by PCR (Platinum Superfi DNA Polymerase, Thermo Fischer Scientific, Waltham, MA, USA) using specific primers harboring either *MluI* or *EcoRI* restriction sequences. This sequence was then cloned into an intermediate plasmid by *MluI*/*EcoRI* restriction/ligation (Thermo Fischer Scientific, Waltham, MA, USA). A dicot codon-optimized construct including the PmCDA1 and the UGI coding sequences was previously synthesized (TwistBioscience, San Francisco, CA, USA) [90] and cloned downstream of the nCas9 sequence through *EcoRI* restriction/ligation (Thermo Fischer Scientific, Waltham, MA, USA). The nCas9_PmCDA1_UGI sequence was cloned into the pDeCas9 backbone through *AscI* restriction/ligation (Thermo Fischer Scientific, Waltham, MA, USA), resulting in the pDenCas9_PmCDA1_UGI vector (Figure 5). The construct was confirmed by Sanger sequencing (Genoscreen, Lille, France). Target sequences in gRNAs were chosen with CRISPOR software 3 [91] (http://crispor.tefor.net/) using the *M. domestica* INRA GGDH13 Version 1.1 genome and NGG PAM. For the *ALS* gene, the same gRNA could be used for both species, because of their DNA and protein sequence similarity on the particular region of the designed guide. For the *PDS* gene, a specific gRNA was chosen for each species. Priory to plant transformation, ‘Gala’ and ‘Conference’ varieties were sequenced for the target sequence to confirm their specificity, using primers listed in Table S1.

Each gRNA cassette marked out by attB gateway sites was synthesized independently by Integrated DNA Technology, Inc. (San Jose, CA, USA) and then cloned in pDONR207 vector by BP cloning (Gateway system; Thermo Fisher Scientific, MA, USA) [92]. Later, the ‘U6gRNA_PDS’ cassette (under MdU6 promoter) was placed after the ‘U3gRNA_ALS’ (under MdU3 promoter) cassette by restriction/ligation at XhoI/PstI sites in the donor vector and SalI/PstI sites in the destination vector, to create pDONR207_U3ALS_U6PDS vector. Gateway LR cloning between pDONR207_U3ALS_U6PDS vector and pDenCas9_PmCDA1_UGI vector was then performed to create pDenMdALSPDS or pDenPcALSPDS (Figure 5). The constructions were confirmed by sequencing of both backbones on the final vector. Sequences are given in File S3.

### 5.3. Plant Transformation

Apple and pear stable transformation were performed on the youngest leaves of 4-week-old microshoots as described in Chevreau et al., 2019 [93]. Briefly, the leaves were vacuum-infiltrated under −0.9 bar during 1 min in a suspension of *A. tumefaciens* (EHA105) at 1 × 10^8^ bacteria/mL and Silwet L-77R (Lehle Seeds, Round Rock, TX, USA) at 0.002% for apple transformation. For pear transformation, a lower bacterial concentration was used (1 × 10^7^ bacteria/mL) and no Silwet L-77R was added to the suspension. The leaves were then wounded transversely with a scalpel. Apple leaves were then transferred to apple regeneration medium consisting of MS medium containing 5 mg/l thidiazuron (TDZ), 0.5 mg/L NAA and 100 mM acetosyringone, solidified with Phytagel TM (Sigma-Aldrich, Saint-Louis, MO, USA) at 3 g/L, while pear leaves were transferred to pear regeneration medium [94] containing 2 mg/L TDZ, 1 mg/L naphthalene acetic acid (NAA) and 100 mM acetosyringone, solidified with Phytagel TM at 3 g/L. Leaves were placed in the dark at 22–24 °C for two days of co-culture with *A. tumefaciens*. For both species, at the end of the co-culture, leaves were placed on their respective regeneration medium containing 300 mg/L cefotaxime, 150 mg/L timentin and 100 mg/L kanamycin. The explants were kept in the dark and the appearance of adventitious buds was monitored every month, along with the transfer to fresh medium for a total of 6 months. All regenerated buds were micropropagated on the same medium as their mother plants, with the addition of 300 mg/L cefotaxime, 150 mg/L timentin, and 100 mg/L kanamycin.

The transgenic plants grew for two months on a medium deprived of chlorsulfuron, to ensure a sufficient development and thereafter the most vigorous lines were transferred to a chlorsulfuron selection medium (1–2 µg/L).

### 5.4. Presence of Transgenes

For the verification of transgene presence in the regenerated plants, genomic DNA was extracted from leaves following Fulton et al., (1995) protocol [95]. The detection of the Cas9 coding sequence, the gRNAs cassette, the *A. tumefaciens* presence, the *nptII* gene and the elongation factor 1a (EF1a) coding gene as a marker of plant DNA suitability were evaluated by PCR with specific and validated primers (Table S1). Amplifications were performed using GoTaq^®^ Flexi DNA Polymerase (Promega, Madison, WI, USA) according to the manufacturer’s recommendations. The PCR reaction conditions were identical for the five genes: 95 °C for 5′, followed by 35 cycles at 95 °C for 30″, 60 °C for 45′, 72 °C for 1′, with a final extension at 72 °C for 5′ and the amplification products were separated for visualization on a 1.5% agarose gel.

### 5.5. Detection of Mutations

For the genomic chimerism removal assay, an initial PCR was performed to amplify the *MdPDS* region from the T0 plant (3E) and from the RT0 regenerated plants (3E1 to 3E12). After, the PCR product was cloned into pGEM^®^-T Easy Vector (Promega, Madison, WI. USA) following the manufacturer’s instructions. Bacterial transformation was performed in One Shot TM TOP10 Chemically Competent *E. coli* and spread on LB plates with 100 mg/mL ampicillin. Colonies were checked by an external PCR, comprising the insert and a part of the destination vector, to confirm fragment insertion. Eight to ten positive clones were sequenced for each line.

For base editing experiment in apple and pear, PCR and sequencing were employed to detect and evaluate the mutations created by each gRNA. For the pear line 75A, leaves were sampled from a totally albino plant. The primers were the same ones used to verify the endogenous sequence gRNA on each variety. These primers amplified a DNA fragment of approximately 260–360 bp for *MdPDS* gene and approximately 224 bp *ALS* (*Malus* and *Pyrus*) surrounding each target. The wild-type apple and pear *ALS* and *PDS* genes fragments were amplified from genomic DNA from ‘Gala’ and ‘Conference’. Bacterial transformation was performed in One-Shot TM TOP10 Chemically Competent *E. coli* and spread on LB plates with 100 mg/mL ampicillin. Colonies were checked by PCR with pUC/M13 primers to confirm fragment insertion. The bacteria-containing putative edited sequences were directly sent for sequencing with four to eight clones sequenced per line. All sequencing results were compared with the reference sequence of their wild type sequence using alignment in MultAlin software [96].

For both experiments, the PCR reactions were performed with the following conditions: 95 °C for 5 min, followed by 35 cycles at 95 °C for 30 s, 60 °C (check primers’ specific temperature on Table S1) for 45 s, 72 °C for 1 min, with a final extension at 72 °C for 5 min. PCR products were cloned into pGEM^®^-T Easy Vector (Promega, Madison, WI, USA) following the manufacturer’s instructions.

Proteins sequence analyses were obtained by translation of DNA sequence into protein with the software by ExPASy Bioinformatic Resource Portal (https://web.expasy.org/translate/) followed by protein alignment at MUltiple Sequence Comparison by Log-Expectation (MUSCLE, https://www.ebi.ac.uk/Tools/msa/muscle/) and visualization on Jalview version 1.8.3–1.1.8 [97].

## Figures and Tables

**Table 1:** Summary of editing profiles of T0 and RT0 transgenic lines for dechimerization assays. The numbers indicate the number of distinct edited alleles versus the total number of wild-type alleles found after the sequencing of 8–10 bacterial clones.

	sgRNA1		sgRNA2		sgRNA1&2	
	Number of Distinct Edited Alleles	Total Number of Wild Sequences	Number of Distinct Edited Alleles	Total Number of Wild Sequences	Number of Distinct Edited Alleles	Total Number of Wild Sequences
T0 line 3E	3	1	2	5	5	1
RT0 line 3E1	5	0	2	5	6	0
RT0 line 3E2	5	0	3	2	7	0
RT0 line 3E3	2	0	1	5	2	0
RT0 line 3E4	2	0	2	1	4	0
RT0 line 3E5	2	0	2	1	4	0
RT0 line 3E6	2	0	3	0	4	0
RT0 line 3E7	3	0	3	4	6	0
RT0 line 3E8	4	0	4	0	4	0
RT0 line 3E9	4	0	3	1	6	0
RT0 line 3E10	2	0	2	0	4	0
RT0 line 3E11	2	0	2	1	4	0
RT0 line 3E12	2	0	1	1	4	0
Total RT0lines analyzed	12		12		12	
Homozygous ^1^ RT0 lines	0		0		0	
Heterozygous ^2^ RT0 lines	0		1 (3E12)		0	
Biallelic ^3^ RT0 lines	7		1		1	
Chimeric ^4^ RT0 lines	5		10		11	
Non edited RT0 lines	0		0		0	

^1^: homozygous = both alleles are mutated and present the same mutation. ^2^: heterozygous = only one allele is mutated.^3^: biallelic = both alleles are mutated but mutations are not identical. ^4^: chimeric = presence of different edited cell line profiles.

**Table 2:** Production of transgenic lines by CBE.

Genotype	Binary Vector	Transformation Experiment	No. Leaves Inoculated	No. Transgenic Lines	Rate of Transformation
‘Gala’	pDenMdALSPDS	N° 323	200	5	2.50%
		N° 338	280	11	3.93%
‘Conference’	pDenPcALSPDS	N° 75	216	2	0.93%

**Table 3:** Summary of editing profiles of transgenic lines CBE.

	pDenMdALS	pDenMdPDS	pDenPcALS	pDenPcPDS
Total lines analyzed	11	11	2	2
Homozygous ^1^ lines	0	1	2	0
Heterozygous ^2^ lines	0	1	0	1
Biallelic ^3^ lines	1	2	0	0
Chimeric ^4^ lines	2	1	0	0
Non edited lines	8	6	0	1
Total clones sequenced	75	69	16	20
Non mutated clones	35	29	0	4

^1^: homozygous = both alleles are mutated and present the same mutation. ^2^: heterozygous = only one allele is mutated.^3^: biallelic = both alleles are mutated but mutations are not identical.^4^: chimeric = presence of different edited cell line profiles.

## Data Availability

Data is contained within the article or supplementary material.

## Supplementary Materials

The following are available online at https://www.mdpi.com/1422-0067/22/1/319/s1, Figure S1. Position of sgRNAs targeting *MdPDS* gene by CRISPR/Cas9 knock-out as published by Charrier et al., 2019. Figure S2. PDS mRNA alleles and derived proteins for apple and pear. Figure S3. Chlorsulfuron sensitivity in apple and pear. File S1. Diversity of allele sequences for PDS protein of RT0 lines after CRISPR/Cas9 and dechimerization strategies. File S2. pDenCas9_PmCDA1_UGI vector coding sequence developed in this study. File S3. *ALS* and *PDS* base editing backbone sequences. Table S1. Primer information concerning dechimerization and base editing assays.

## Abbreviations

BE: base editing; ABE, adenine base editing; CBE, cytosine base editing; AID, activation-induced cytidine deaminase; CRISPR, clustered regularly interspaced short palindromic repeats; dCas9, dead Cas9; nCas9, nickase Cas9; DSBs, double-strand breaks; HDR, homology-directed repair; Indels, insertions and deletions; KO, Knock-out; NHEJ, non-homologous end-joining; PAM, protospacer adjacent motif; sgRNA, single-guide RNA; UGI, uracil DNA glycosylase inhibitor.
