# Supplementary material for: New Strategies to Overcome Present CRISPR/Cas9 Limitations in Apple and Pear: Efficient Dechimerization and Base Editing
Source: Int J Mol Sci. 2020 Dec 30;22(1):319. doi: 10.3390/ijms22010319 (PMC7795782; doi:10.3390/ijms22010319)
Supplement: Supplementary file 1 [file ijms-22-00319-s001.zip › supplementary/Figure S1.pdf]

Supplementary material

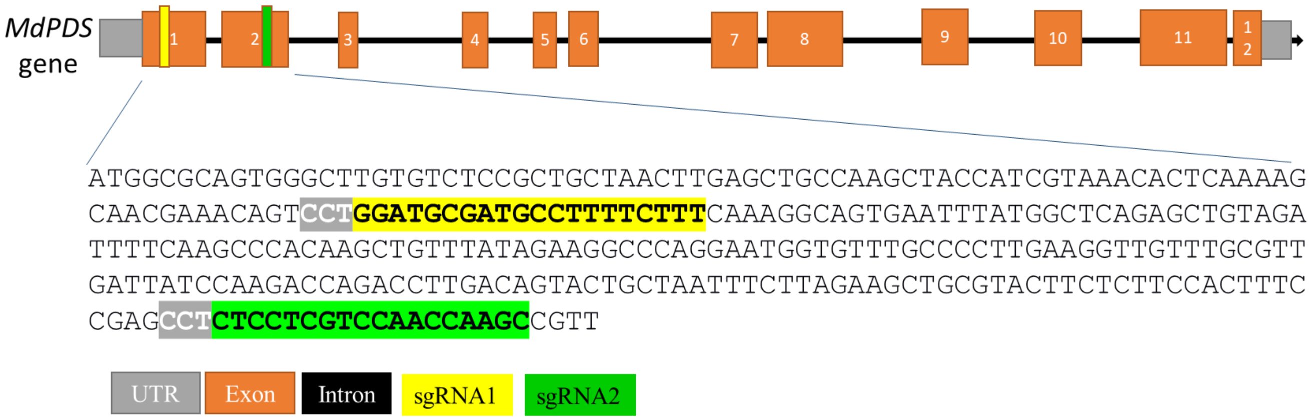

Figure S1. Position of sgRNAs targeting *MdPDS* gene by CRISPR/Cas9 knock-out as published by Charrier et al., 2019. *MdPDS* gene complete sequence illustrated with UTRs (gray boxes), exons (orange boxes) and introns (black lines). sgRNAs designed by Charrier et al., 2019 are indicated. Single guide RNA1 located on the first exon is marked in yellow and single guide RNA 2 located at the second exon is marked in green. The PAM sequences for both sgRNAs are marked in gray (CCT).
