# Supplementary material for: New Strategies to Overcome Present CRISPR/Cas9 Limitations in Apple and Pear: Efficient Dechimerization and Base Editing
Source: Int J Mol Sci. 2020 Dec 30;22(1):319. doi: 10.3390/ijms22010319 (PMC7795782; doi:10.3390/ijms22010319)
Supplement: Supplementary file 1 [file ijms-22-00319-s001.zip › supplementary/Figure S3.pdf]

Supplementary material

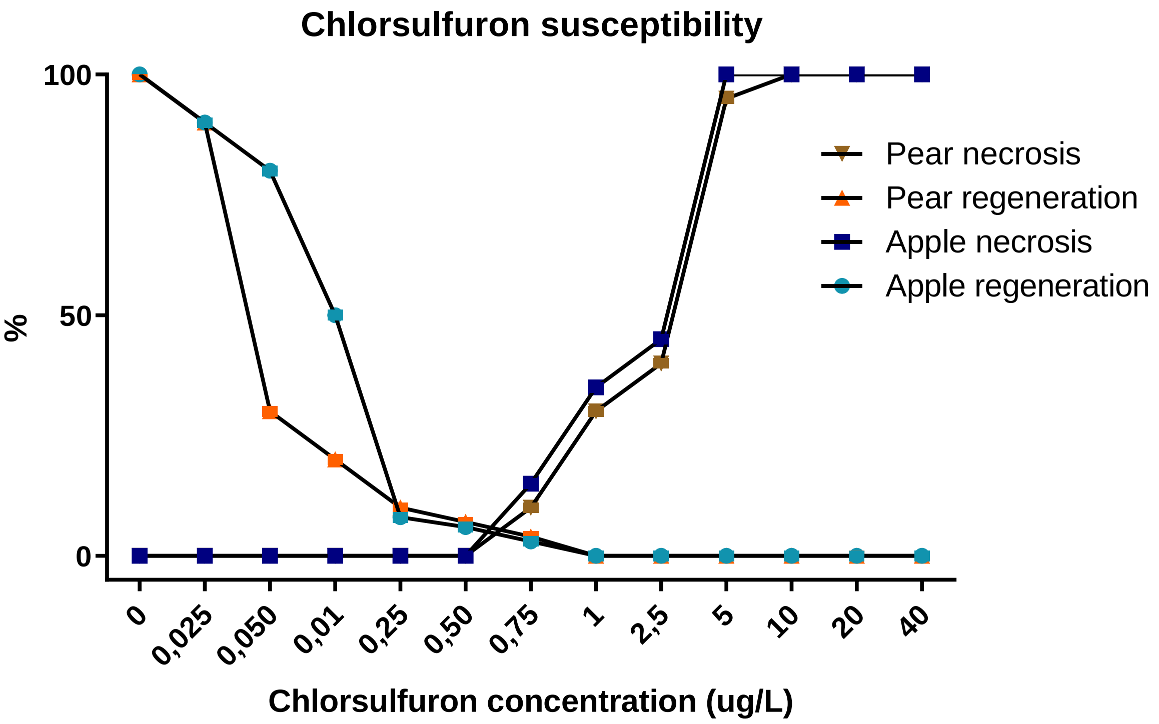

Figure S3. Chlorsulfuron susceptibility in apple and pear. Range of chlorsulfuron concentration applied to regeneration medium in order to observe bud formation and necrosis on leaves.
