# Supplementary material for: New Strategies to Overcome Present CRISPR/Cas9 Limitations in Apple and Pear: Efficient Dechimerization and Base Editing
Source: Int J Mol Sci. 2020 Dec 30;22(1):319. doi: 10.3390/ijms22010319 (PMC7795782; doi:10.3390/ijms22010319)
Supplement: Supplementary file 1 [file ijms-22-00319-s001.zip › supplementary/File S1.pdf]

Supplementary material

File 1.

Diversity of allele sequences for PDS protein of RT0 lines after CRISPR/Cas9 and dechimerization strategies. Dash (-) means a stop codon was detected.

```
>NATIVE
MAQWACVSAANLSCQATIVNTQKQRNSPGCDAFSFKGSEFMAQSCRFSSPQAVYRRPRNGVCPLKVVCVDYPRPD
LDSTANFLEAAYFSSTFRASPRPTKPLKVVIAGAGLAGLATAKYLADAGHQPILLEARDVLGGKDGGLGIIELSA
FDQTLLSVGGGVCVGMGDVLSDRWQHGKIVMGTGMKQACIYSERMMECNSVTNLGMRFLVVSSLHYSPSSVWFKV
QTVPGKFKVAVPNILGVRTPGFPASVFIVIFCPRWIYIIYTLLNETGFGAYPNIQNLFGELGINDRLQWKEHSMI
FAMPNKPGEFSRFDFLEVLPAPINGKYYSHLLKIESSLFTNAENSFCSWEGIWAILKNNEMLTWPEKIKFAIGLL
PAILGGQAYVEAQDGLSVKDWMRKQGIPDRVTTEVFIAMSKALNFINPDELSMQCILIALNRFLQEKHGSKMAFL
DGSPPERLCAPIVDHIQSLGGEVRTNSRIQKIDLNNDGTVKSFVLNNGSVIEADAYVFATPVDILKLLLPENWKE
MPYFKKLEKLVGVPVINVHIW-

>T101
MAQWACVSAANLSCQATIVNTQKQRNSPGCLFFQRQ-IYGSEL-IFKPTSCL-KAQEWCLPLEGCLR-LSKTRP-
QYC-FLRSCVLLFHFPSLSLVQPSR-KL-LLVQVWLVWQLQNIWRMRVINLYY-KREMF-AERMVGWELLS-
VLLIRHSYLLVAGCVLGWGMCCLTGGSMER--WGLV-NRPAYILRG-WSVTV-QIWE-DS--
LVACIILLPVFGSKFRLCQENLR-LYLTS-
GFEPQDFQHLFSLSSSVQGGSTLFTHCLMKLVLGHIQIFRICLESLVLTIGCSGRNIL-
YLQCQTSQGSSVGLISWKFCQHP-MVNITVTS-RLKVVCLQMQKILSVPGKEYGPY-RTMRC-
LGQRKSSLQLDYCQQSLVGRLMLKPKMA-A-KTG-GNRAYLIE-LQRCL-PCQRPLTLLTLMNFQCSAY-LL-
TDSSRRNTVPRWLSWMVVPPRDSVLQLLIISSHWAVKSELIPEYRKLI-ITMEL-RVLY-IMGA-
LKQMRMCSPLQLIS-SFYCLKTGKRCHISRNWRN-LEFQLSMFTYG

>T102
MAQWACVSAANLSCQATIVNTQKQRNSPGSMPFLSKAVNLWLRAVDFQAHKLFIEGPGMVFAP-
RLFALIIQDQTLTVLLIS-KLRTSLPLSEPLLVQPSR-KL-LLVQVWLVWQLQNIWRMRVINLYY-KREMF-
AERMVGWELLS-VLLIRHSYLLVAGCVLGWGMCCLTGGSMER--WGLV-NRPAYILRG-WSVTV-QIWE-DS--
LVACIILLPVFGSKFRLCQENLR-LYLTS-
GFEPQDFQHLFSLSSSVQGGSTLFTHCLMKLVLGHIQIFRICLESLVLTIGCSGRNIL-
YLQCQTSQGSSVGLISWKFCQHP-MVNITVTS-RLKVVCLQMQKILSVPGKEYGPY-RTMRC-
LGQRKSSLQLDYCQQSLVGRLMLKPKMA-A-KTG-GNRAYLIE-LQRCL-PCQRPLTLLTLMNFQCSAY-LL-
TDSSRRNTVPRWLSWMVVPPRDSVLQLLIISSHWAVKSELIPEYRKLI-ITMEL-RVLY-IMGA-
LKQMRMCSPLQLIS-SFYCLKTGKRCHISRNWRN-LEFQLSMFTYG
```

>T103

MAQWACVSAANLSCQATIVNTQKQRNSPGSMPFLSKAVNLWLRAVDFQAHKLFIEGPGMVFAP-

RLFALIIQDQTLTVLLIS-

KLRTSLPLSEPLPRPTKPLKVVIAGAGLAGLATAKYLADAGHQPILLEARDVLGGKDGGLGIIELSAFDQTLLSV

GGGVCVGMGDVLSDRWQHGKIVMGTGMKQACIYSERMMECNSVTNLGMRFLVVSSLHYSPSSVWFKVQTVPGKFK

VAVPNILGVRTPGFPASVFIVIFCPRWIYIIYTLLNETGFGAYPNIQNLFGELGINDRLQWKEHSMIFAMPNKPG

EFSRFDFLEVLPAPINGKYYSHLLKIESSLFTNAENSFCSWEGIWAILKNNEMLTWPEKIKFAIGLLPAILGGQA

YVEAQDGLSVKDWMRKQGIPDRVTTEVFIAMSKALNFINPDELSMQCILIALNRFLQEKHGSKMAFLDGSPPERL

CAPIVDHIQSLGGEVRTNSRIQKIDLNNDGTVKSFVLNNGSVIEADAYVFATPVDILKLLLPENWKEMPYFKKLE

KLVGVPVINVHIW-

>T104

MAQWACVSAANLSCQATIVNTQKQRNSPGLRCLFFQRQ-IYGSEL-IFKPTSCL-KAQEWCLPLEGCLR-

LSKTRP-QYC-FLRSCVLLFHFPSLSLVQPSR-KL-LLVQVWLVWQLQNIWRMRVINLYY-KREMF-

AERMVGWELLS-VLLIRHSYLLVAGCVLGWGMCCLTGGSMER--WGLV-NRPAYILRG-WSVTV-QIWE-DS--

LVACIILLPVFGSKFRLCQENLR-LYLTS-

GFEPQDFQHLFSLSSSVQGGSTLFTHCLMKLVLGHIQIFRICLESLVLTIGCSGRNIL-

YLQCQTSQGSSVGLISWKFCQHP-MVNITVTS-RLKVVCLQMQKILSVPGKEYGPY-RTMRC-

LGQRKSSLQLDYCQQSLVGRLMLKPKMA-A-KTG-GNRAYLIE-LQRCL-PCQRPLTLLTLMNFQCSAY-LL-

TDSSRRNTVPRWLSWMVVPPRDSVLQLLIISSHWAVKSELIPEYRKLI-ITMEL-RVLY-IMGA-

LKQMRMCSPLQLIS-SFYCLKTGKRCHISRNWRN-LEFQLSMFTYG

>T105

MAQWACVSAANLSCQATIVNTQKQRNSPGCLFFQRQ-IYGSEL-IFKPTSCL-KAQEWCLPLEGCLR-LSKTRP-

QYC-FLRSCVLLFHFPSLSLVQPSR-KL-LLVQVWLVWQLQNIWRMRVINLYY-KREMF-AERMVGWELLS-

VLLIRHSYLLVAGCVLGWGMCCLTGGSMER--WGLV-NRPAYILRG-WSVTV-QIWE-DS--

LVACIILLPVFGSKFRLCQENLR-LYLTS-

GFEPQDFQHLFSLSSSVQGGSTLFTHCLMKLVLGHIQIFRICLESLVLTIGCSGRNIL-

YLQCQTSQGSSVGLISWKFCQHP-MVNITVTS-RLKVVCLQMQKILSVPGKEYGPY-RTMRC-

LGQRKSSLQLDYCQQSLVGRLMLKPKMA-A-KTG-GNRAYLIE-LQRCL-PCQRPLTLLTLMNFQCSAY-LL-

TDSSRRNTVPRWLSWMVVPPRDSVLQLLIISSHWAVKSELIPEYRKLI-ITMEL-RVLY-IMGA-

LKQMRMCSPLQLIS-SFYCLKTGKRCHISRNWRN-LEFQLSMFTYG

>T106

MAQWACVSAANLSCQATIVNTQKQRNSPGSSNQAVKSCDCWCRFGWSGNCKIFGGCGSSTYTTRSERCFRRKGWW

VGNY-VECF-SDTLICWWRGVCWDGGCVV-QVAAWKDSDGDWYETGLHIF-EDDGV-QCNKSGNEIPSS--

LALFSFQCLVQSSDCARKI-GSCT-HLRGSNPRISSICFHCHLLSKVDLHYLHIA--

NWFWGISKYSESVWRAWY-RSVAVEGTFYDICNAKQARGVQSV-FPGSSASTHKW-ILQSPLED-K-

FVYKCRKFFLFLGRNMGHIEEQ-DADLARENQVCNWTTASNPWWAGLC-SPRWLERKRLDEETGHT-

SSNYRGVYSHVKGP-LY-P--TFNAVHIDCFEPIPPGETRFQDGFLGW-SPRETLCSNC-SYPVIGR-SPN-

FPNTEN-SK-RWNCEEFCTK-WERD-SRCVCVRHSS-YLKASIA-KLERDAIFQEIGEISWSSSYQCSHMV

>T107

MAQWACVSAANLSCQATIVNTQKQRNSPGSMPFLSKAVNLWLRAVDFQAHKLFIEGPGMVFAP-
RLFALIIQDQTLTVLLIS-
KLRTSLPLSEPLLVQPTKPLKVVIAGAGLAGLATAKYLADAGHQPILLEARDVLGGKDGGLGIIELSAFDQTLLS
VGGGVCVGMGDVLSDRWQHGKIVMGTGMKQACIYSERMMECNSVTNLGMRFLVVSSLHYSPSSVWFKVQTVPGKF
KVAVPNILGVRTPGFPASVFIVIFCPRWIYIIYTLLNETGFGAYPNIQNLFGELGINDRLQWKEHSMIFAMPNKP
GEFSRFDFLEVLPAPINGKYYSHLLKIESSLFTNAENSFCSWEGIWAILKNNEMLTWPEKIKFAIGLLPAILGGQ
AYVEAQDGLSVKDWMRKQGIPDRVTTEVFIAMSKALNFINPDELSMQCILIALNRFLQEKHGSKMAFLDGSPPER
LCAPIVDHIQSLGGEVRTNSRIQKIDLNNDGTVKSFVLNNGSVIEADAYVFATPVDILKLLLPENWKEMPYFKKL
EKLVGVPVINVHIW-

>T108

MAQWACVSAANLSCQATIVNTQKQRNSPGLRCLFFQRQ-IYGSEL-IFKPTSCL-KAQEWCLPLEGCLR-
LSKTRP-QYC-
FLRSCVLLFHFPSLSSNQAVKSCDCWCRFGWSGNCKIFGGCGSSTYTTRSERCFRRKGWWVGNY-VECF-
SDTLICWWRGVCWDGGCVV-QVAAWKDSDGDWYETGLHIF-EDDGV-QCNKSGNEIPSS--
LALFSFQCLVQSSDCARKI-GSCT-HLRGSNPRISSICFHCHLLSKVDLHYLHIA--
NWFWGISKYSESVWRAWY-RSVAVEGTFYDICNAKQARGVQSV-FPGSSASTHKW-ILQSPLED-K-
FVYKCRKFFLFLGRNMGHIEEQ-DADLARENQVCNWTTASNPWWAGLC-SPRWLERKRLDEETGHT-
SSNYRGVYSHVKGP-LY-P--TFNAVHIDCFEPIPPGETRFQDGFLGW-SPRETLCSNC-SYPVIGR-SPN-
FPNTEN-SK-RWNCEEFCTK-WERD-SRCVCVRHSS-YLKASIA-KLERDAIFQEIGEISWSSSYQCSHMV

>T109

MAQWACVSAANLSCQATIVNTQKQRNSPGCLFFQRQ-IYGSEL-IFKPTSCL-KAQEWCLPLEGCLR-LSKTRP-
QYC-FLRSCVLLFHFPSLSSSNQAVKSCDCWCRFGWSGNCKIFGGCGSSTYTTRSERCFRRKGWWVGNY-VECF-
SDTLICWWRGVCWDGGCVV-QVAAWKDSDGDWYETGLHIF-EDDGV-QCNKSGNEIPSS--
LALFSFQCLVQSSDCARKI-GSCT-HLRGSNPRISSICFHCHLLSKVDLHYLHIA--
NWFWGISKYSESVWRAWY-RSVAVEGTFYDICNAKQARGVQSV-FPGSSASTHKW-ILQSPLED-K-
FVYKCRKFFLFLGRNMGHIEEQ-DADLARENQVCNWTTASNPWWAGLC-SPRWLERKRLDEETGHT-
SSNYRGVYSHVKGP-LY-P--TFNAVHIDCFEPIPPGETRFQDGFLGW-SPRETLCSNC-SYPVIGR-SPN-
FPNTEN-SK-RWNCEEFCTK-WERD-SRCVCVRHSS-YLKASIA-KLERDAIFQEIGEISWSSSYQCSHMV

>T1010

MAQWACVSAANLSCQATIVNTQKQRNSPAFSFKGSEFMAQSCRFSSPQAVYRRPRNGVCPLKVVCVDYPRPDLDS
TANFLEAAYFSSTFRASPRPTKPLKVVIAGAGLAGLATAKYLADAGHQPILLEARDVLGGKDGGLGIIELSAFDQ
TLLSVGGGVCVGMGDVLSDRWQHGKIVMGTGMKQACIYSERMMECNSVTNLGMRFLVVSSLHYSPSSVWFKVQTV
PGKFKVAVPNILGVRTPGFPASVFIVIFCPRWIYIIYTLLNETGFGAYPNIQNLFGELGINDRLQWKEHSMIFAM
PNKPGEFSRFDFLEVLPAPINGKYYSHLLKIESSLFTNAENSFCSWEGIWAILKNNEMLTWPEKIKFAIGLLPAI
LGGQAYVEAQDGLSVKDWMRKQGIPDRVTTEVFIAMSKALNFINPDELSMQCILIALNRFLQEKHGSKMAFLDGS

PPERLCAPIVDHIQSLGGEVRTNSRIQKIDLNNDGTVKSFVLNNGSVIEADAYVFATPVDILKLLLPENWKEMPY
FKKLEKLVGVPVINVHIW-

>T1011
MAQWACVSAANLSCQATIVNTQKQRNSPGLRCLFFQRQ-IYGSEL-IFKPTSCL-KAQEWCLPLEGCLR-
LSKTRP-QYC-
FLRSCVLLFHFPSSPRPTKPLKVVIAGAGLAGLATAKYLADAGHQPILLEARDVLGGKDGGLGIIELSAFDQTLL
SVGGGVCVGMGDVLSDRWQHGKIVMGTGMKQACIYSERMMECNSVTNLGMRFLVVSSLHYSPSSVWFKVQTVPGK
FKVAVPNILGVRTPGFPASVFIVIFCPRWIYIIYTLLNETGFGAYPNIQNLFGELGINDRLQWKEHSMIFAMPNK
PGEFSRFDFLEVLPAPINGKYYSHLLKIESSLFTNAENSFCSWEGIWAILKNNEMLTWPEKIKFAIGLLPAILGG
QAYVEAQDGLSVKDWMRKQGIPDRVTTEVFIAMSKALNFINPDELSMQCILIALNRFLQEKHGSKMAFLDGSPPE
RLCAPIVDHIQSLGGEVRTNSRIQKIDLNNDGTVKSFVLNNGSVIEADAYVFATPVDILKLLLPENWKEMPYFKK
LEKLVGVPVINVHIW-

>T1012
MAQWACVSAANLSCQATIVNTQKQRNSPGSMPFLSKAVNLWLRAVDFQAHKLFIEGPGMVFAP-
RLFALIIQDQTLTVLLIS-
KLRTSLPLSEPLSSNQAVKSCDCWCRFGWSGNCKIFGGCGSSTYTTRSERCFRRKGWWVGNY-VECF-
SDTLICWWRGVCWDGGCVV-QVAAWKDSDGDWYETGLHIF-EDDGV-QCNKSGNEIPSS--
LALFSFQCLVQSSDCARKI-GSCT-HLRGSNPRISSICFHCHLLSKVDLHYLHIA--
NWFWGISKYSESVWRAWY-RSVAVEGTFYDICNAKQARGVQSV-FPGSSASTHKW-ILQSPLED-K-
FVYKCRKFFLFLGRNMGHIEEQ-DADLARENQVCNWTTASNPWWAGLC-SPRWLERKRLDEETGHT-
SSNYRGVYSHVKGP-LY-P--TFNAVHIDCFEPIPPGETRFQDGFLGW-SPRETLCSNC-SYPVIGR-SPN-
FPNTEN-SK-RWNCEEFCTK-WERD-SRCVCVRHSS-YLKASIA-KLERDAIFQEIGEISWSSSYQCSHMV

>T1013
MAQWACVSAANLSCQATIVNTQKQRNSPGAMPFLSKAVNLWLRAVDFQAHKLFIEGPGMVFAP-
RLFALIIQDQTLTVLLIS-
KLRTSLPLSEPLTRPTKPLKVVIAGAGLAGLATAKYLADAGHQPILLEARDVLGGKDGGLGIIELSAFDQTLLSV
GGGVCVGMGDVLSDRWQHGKIVMGTGMKQACIYSERMMECNSVTNLGMRFLVVSSLHYSPSSVWFKVQTVPGKFK
VAVPNILGVRTPGFPASVFIVIFCPRWIYIIYTLLNETGFGAYPNIQNLFGELGINDRLQWKEHSMIFAMPNKPG
EFSRFDFLEVLPAPINGKYYSHLLKIESSLFTNAENSFCSWEGIWAILKNNEMLTWPEKIKFAIGLLPAILGGQA
YVEAQDGLSVKDWMRKQGIPDRVTTEVFIAMSKALNFINPDELSMQCILIALNRFLQEKHGSKMAFLDGSPPERL
CAPIVDHIQSLGGEVRTNSRIQKIDLNNDGTVKSFVLNNGSVIEADAYVFATPVDILKLLLPENWKEMPYFKKLE
KLVGVPVINVHIW-

>T1014
MAQWACVSAANLSCQATIVNTQKQRNSPGLFFQRQ-IYGSEL-IFKPTSCL-KAQEWCLPLEGCLR-LSKTRP-
QYC-FLRSCVLLFHFPSLSSSNQAVKSCDCWCRFGWSGNCKIFGGCGSSTYTTRSERCFRRKGWWVGNY-VECF-
SDTLICWWRGVCWDGGCVV-QVAAWKDSDGDWYETGLHIF-EDDGV-QCNKSGNEIPSS--
LALFSFQCLVQSSDCARKI-GSCT-HLRGSNPRISSICFHCHLLSKVDLHYLHIA--

NWFWGISKYSESVWRAWY-RSVAVEGTFYDICNAKQARGVQSV-FPGSSASTHKW-ILQSPLED-K-
FVYKCRKFFLFLGRNMGHIEEQ-DADLARENQVCNWTTASNPWWAGLC-SPRWLERKRLDEETGHT-
SSNYRGVYSHVKGP-LY-P--TFNAVHIDCFEPIPPGETRFQDGFLGW-SPRETLCSNC-SYPVIGR-SPN-
FPNTEN-SK-RWNCEEFCTK-WERD-SRCVCVRHSS-YLKASIA-KLERDAIFQEIGEISWSSSYQCSHMV

>T1015
MAQWACVSAANLSCQATIVNTQKQRNSPGCLFFQRQ-IYGSEL-IFKPTSCL-KAQEWCLPLEGCLR-LSKTRP-
QYC-
FLRSCVLLFHFPSLSRPTKPLKVVIAGAGLAGLATAKYLADAGHQPILLEARDVLGGKDGGLGIIELSAFDQTLL
SVGGGVCVGMGDVLSDRWQHGKIVMGTGMKQACIYSERMMECNSVTNLGMRFLVVSSLHYSPSSVWFKVQTVPGK
FKVAVPNILGVRTPGFPASVFIVIFCPRWIYIIYTLLNETGFGAYPNIQNLFGELGINDRLQWKEHSMIFAMPNK
PGEFSRFDFLEVLPAPINGKYYSHLLKIESSLFTNAENSFCSWEGIWAILKNNEMLTWPEKIKFAIGLLPAILGG
QAYVEAQDGLSVKDWMRKQGIPDRVTTEVFIAMSKALNFINPDELSMQCILIALNRFLQEKHGSKMAFLDGSPPE
RLCAPIVDHIQSLGGEVRTNSRIQKIDLNNDGTVKSFVLNNGSVIEADAYVFATPVDILKLLLPENWKEMPYFKK
LEKLVGVPVINVHIW-

>T1016
MAQWACVSAANLSCQATIVNTQKQRNSPGCLFFQRQ-IYGSEL-IFKPTSCL-KAQEWCLPLEGCLR-LSKTRP-
QYC-FLRSCVLLFHFPSLSSSNQPVKSCDCWCRFGWSGNCKIFGGCGSSTYTTRSERCFRRKGWWVGNY-VECF-
SDTLICWWRGVCWDGGCVV-QVAAWKDSDGDWYETGLHIF-EDDGV-QCNKSGNEIPSS--
LALFSFQCLVQSSDCARKI-GSCT-HLRGSNPRISSICFHCHLLSKVDLHYLHIA--
NWFWGISKYSESVWRAWY-RSVAVEGTFYDICNAKQARGVQSV-FPGSSASTHKW-ILQSPLED-K-
FVYKCRKFFLFLGRNMGHIEEQ-DADLARENQVCNWTTASNPWWAGLC-SPRWLERKRLDEETGHT-
SSNYRGVYSHVKGP-LY-P--TFNAVHIDCFEPIPPGETRFQDGFLGW-SPRETLCSNC-SYPVIGR-SPN-
FPNTEN-SK-RWNCEEFCTK-WERD-SRCVCVRHSS-YLKASIA-KLERDAIFQEIGEISWSSSYQCSHMV

>T1017
MAQWACVSAANLSCQATIVNTQKQRNSPGLRCLFFQRQ-IYGSEL-IFKPTSCL-KAQEWCLPLEGCLR-
LSKTRP-QYC-
FLRSCVLLFHFPSLSSNQAVKSCDCWCRFGWSGNCKIFGGCGSSTYTTRSERCFRRKGWWVGNY-VECF-
SDTLICWWRGVCWDGGCVV-QVAAWKDSDGDWYETGLHIF-EDDGV-QCNKSGNEIPSS--
LALFSFQCLVQSSDCARKI-GSCT-HLRGSNPRISSICFHCHLLSKVDLHYLHIA--
NWFWGISKYSESVWRAWY-RSVAVEGTFYDICNAKQARGVQSV-FPGSSASTHKW-ILQSPLED-K-
FVYKCRKFFLFLGRNMGHIEEQ-DADLARENQVCNWTTASNPWWAGLC-SPRWLERKRLDEETGHT-
SSNYRGVYSHVKGP-LY-P--TFNAVHIDCFEPIPPGETRFQDGFLGW-SPRETLCSNC-SYPVIGR-SPN-
FPNTEN-SK-RWNCEEFCTK-WERD-SRCVCVRHSS-YLKASIA-KLERDAIFQEIGEISWSSSYQCSHMV

>T1018
MAQWACVSAANLSCQATIVNTQKQRNSPGCPFLSKAVNLWLRAVDFQAHKLFIEGPGMVFAP-
RLFALIIQDQTLTVLLIS-
KLRTSLPLSEPLTRPTKPLKVVIAGAGLAGLATAKYLADAGHQPILLEARDVLGGKDGGLGIIELSAFDQTLLSV

GGGVCVGMGDVLSDRWQHGKIVMGTGMKQACIYSERMMECNSVTNLGMRFLVVSSLHYSPSSVWFKVQTVPGKFK
VAVPNILGVRTPGFPASVFIVIFCPRWIYIIYTLLNETGFGAYPNIQNLFGELGINDRLQWKEHSMIFAMPNKPG
EFSRFDFLEVLPAPINGKYYSHLLKIESSLFTNAENSFCSWEGIWAILKNNEMLTWPEKIKFAIGLLPAILGGQA
YVEAQDGLSVKDWMRKQGIPDRVTTEVFIAMSKALNFINPDELSMQCILIALNRFLQEKHGSKMAFLDGSPPERL
CAPIVDHIQSLGGEVRTNSRIQKIDLNNDGTVKSFVLNNGSVIEADAYVFATPVDILKLLLPENWKEMPYFKKLE
KLVGVPVINVHIW-

>T1019
MAQWACVSAANLSCQATIVNTQKQRNSPGCLFFQRQ-IYGSEL-IFKPTSCL-KAQEWCLPLEGCLR-LSKTRP-
QYC-FLRSCVLLFHFPSLSSNQAVKSCDCWCRFGWSGNCKIFGGCGSSTYTTRSERCFRRKGWWVGNY-VECF-
SDTLICWWRGVCWDGGCVV-QVAAWKDSDGDWYETGLHIF-EDDGV-QCNKSGNEIPSS--
LALFSFQCLVQSSDCARKI-GSCT-HLRGSNPRISSICFHCHLLSKVDLHYLHIA--
NWFWGISKYSESVWRAWY-RSVAVEGTFYDICNAKQARGVQSV-FPGSSASTHKW-ILQSPLED-K-
FVYKCRKFFLFLGRNMGHIEEQ-DADLARENQVCNWTTASNPWWAGLC-SPRWLERKRLDEETGHT-
SSNYRGVYSHVKGP-LY-P--TFNAVHIDCFEPIPPGETRFQDGFLGW-SPRETLCSNC-SYPVIGR-SPN-
FPNTEN-SK-RWNCEEFCTK-WERD-SRCVCVRHSS-YLKASIA-KLERDAIFQEIGEISWSSSYQCSHMV

>T1020
MAQWACVSAANLSCQATIVNTQKQRNSPGCLFSQRQ-IYGSEL-IFKPTSCL-KAQEWCLPLEGCLR-LSKTRP-
QYC-FLRSCVLLFHFPSLSLVQPSR-KL-LLVQVWLVWQLQNIWRMRVINLYY-KREMF-AERMVGWELLS-
VLLIRHSYLLVAGCVLGWGMCCLTGGSMER--WGLV-NRPAYILRG-WSVTV-QIWE-DS--
LVACIILLPVFGSKFRLCQENLR-LYLTS-
GFEPQDFQHLFSLSSSVQGGSTLFTHCLMKLVLGHIQIFRICLESLVLTIGCSGRNIL-
YLQCQTSQGSSVGLISWKFCQHP-MVNITVTS-RLKVVCLQMQKILSVPGKEYGPY-RTMRC-
LGQRKSSLQLDYCQQSLVGRLMLKPKMA-A-KTG-GNRAYLIE-LQRCL-PCQRPLTLLTLMNFQCSAY-LL-
TDSSRRNTVPRWLSWMVVPPRDSVLQLLIISSHWAVKSELIPEYRKLI-ITMEL-RVLY-IMGA-
LKQMRMCSPLQLIS-SFYCLKTGKRCHISRNWRN-LEFQLSMFTYG

>T1021
MAQWACVSAANLSCQATIVNTQKQRNSPGLRCLFFQRQ-IYGSEL-IFKPTSCL-KAQEWCLPLEGCLR-
LSKTRP-QYC-FLRSCVLLFHFPSLSLVQPSR-KL-LLVQVWLVWQLQNIWRMRVINLYY-KREMF-
AERMVGWELLS-VLLIRHSYLLVAGCVLGWGMCCLTGGSMER--WGLV-NRPAYILRG-WSVTV-QIWE-DS--
LVACIILLPVFGSKFRLCQENLR-LYLTS-
GFEPQDFQHLFSLSSSVQGGSTLFTHCLMKLVLGHIQIFRICLESLVLTIGCSGRNIL-
YLQCQTSQGSSVGLISWKFCQHP-MVNITVTS-RLKVVCLQMQKILSVPGKEYGPY-RTMRC-
LGQRKSSLQLDYCQQSLVGRLMLKPKMA-A-KTG-GNRAYLIE-LQRCL-PCQRPLTLLTLMNFQCSAY-LL-
TDSSRRNTVPRWLSWMVVPPRDSVLQLLIISSHWAVKSELIPEYRKLI-ITMEL-RVLY-IMGA-
LKQMRMCSPLQLIS-SFYCLKTGKRCHISRNWRN-LEFQLSMFTYG

>T1022

MAQWACVSAANLSCQATIVNTQKQRNSPGFAMPFLSKAVNLWLRAVDFQAHKLFIEGPGMVFAP-
RLFALIIQDQTLTVLLIS-
KLRTSLPLSEPSPRPTKPLKVVIAGAGLAGLATAKYLADAGHQPILLEARDVLGGKDGGLGIIELSAFDQTLLSV
GGGVCVGMGDVLSDRWQHGKIVMGTGMKQACIYSERMMECNSVTNLGMRFLVVSSLHYSPSSVWFKVQTVPGKFK
VAVPNILGVRTPGFPASVFIVIFCPRWIYIIYTLLNETGFGAYPNIQNLFGELGINDRLQWKEHSMIFAMPNKPG
EFSRFDFLEVLPAPINGKYYSHLLKIESSLFTNAENSFCSWEGIWAILKNNEMLTWPEKIKFAIGLLPAILGGQA
YVEAQDGLSVKDWMRKQGIPDRVTTEVFIAMSKALNFINPDELSMQCILIALNRFLQEKHGSKMAFLDGSPPERL
CAPIVDHIQSLGGEVRTNSRIQKIDLNNDGTVKSFVLNNGSVIEADAYVFATPVDILKLLLPENWKEMPYFKKLE
KLVGVPVINVHIW-

>T1023
MAQWACVSAANLSCQATIVNTQKQRNSPGCHFFQRQ-IYGSEL-IFKPTSCL-KAQEWCLPLEGCLR-LSKTRP-
QYC-FLRSCVLLFHFPSLSLVQPSSR-KL-LLVQVWLVWQLQNIWRMRVINLYY-KREMF-AERMVGWELLS-
VLLIRHSYLLVAGCVLGWGMCCLTGGSMER--WGLV-NRPAYILRG-WSVTV-QIWE-DS--
LVACIILLPVFGSKFRLCQENLR-LYLTS-
GFEPQDFQHLFSLSSSVQGGSTLFTHCLMKLVLGHIQIFRICLESLVLTIGCSGRNIL-
YLQCQTSQGSSVGLISWKFCQHP-MVNITVTS-RLKVVCLQMQKILSVPGKEYGPY-RTMRC-
LGQRKSSLQLDYCQQSLVGRLMLKPKMA-A-KTG-GNRAYLIE-LQRCL-PCQRPLTLLTLMNFQCSAY-LL-
TDSSRRNTVPRWLSWMVVPPRDSVLQLLIISSHWAVKSELIPEYRKLI-ITMEL-RVLY-IMGA-
LKQMRMCSPLQLIS-SFYCLKTGKRCHISRNWRN-LEFQLSMFTYG

>T1024
MAQWACVSAANLSCQATIVNTQKQRNSPGSMPFLSKAVNLWLRAVDFQAHKLFIEGPGMVFAP-
RLFALIIQDQTLTVLLIS-
KLRTSLPLSEPLTRPTKPLKVVIAGAGLAGLATAKYLADAGHQPILLEARDVLGGKDGGLGIIELSAFDQTLLSV
GGGVCVGMGDVLSDRWQHGKIVMGTGMKQACIYSERMMECNSVTNLGMRFLVVSSLHYSPSSVWFKVQTVPGKFK
VAVPNILGVRTPGFPASVFIVIFCPRWIYIIYTLLNETGFGAYPNIQNLFGELGINDRLQWKEHSMIFAMPNKPG
EFSRFDFLEVLPAPINGKYYSHLLKIESSLFTNAENSFCSWEGIWAILKNNEMLTWPEKIKFAIGLLPAILGGQA
YVEAQDGLSVKDWMRKQGIPDRVTTEVFIAMSKALNFINPDELSMQCILIALNRFLQEKHGSKMAFLDGSPPERL
CAPIVDHIQSLGGEVRTNSRIQKIDLNNDGTVKSFVLNNGSVIEADAYVFATPVDILKLLLPENWKEMPYFKKLE
KLVGVPVINVHIW-

>T1025
MAQWACVSAANLSCQATIVNTQKQRNSPGCLFFQRQ-IYGSEL-IFKPTSCL-KAQEWCLPLEGCLR-LSKTRP-
QYC-FLRSCVLLFHFPSLSLVQPSR-KL-LLVQVWLVWQLQNIWRMRVINLYY-KREMF-AERMVGWELLS-
VLLIRHSYLLVAGCVLGWGMCCLTGGSMER--WGLV-NRPAYILRG-WSVTV-QIWE-DS--
LVACIILLPVFGSKFRLCQENLR-LYLTS-
GFEPQDFQHLFSLSSSVQGGSTLFTHCLMKLVLGHIQIFRICLESLVLTIGCSGRNIL-
YLQCQTSQGSSVGLISWKFCQHP-MVNITVTS-RLKVVCLQMQKILSVPGKEYGPY-RTMRC-
LGQRKSSLQLDYCQQSLVGRLMLKPKMA-A-KTG-GNRAYLIE-LQRCL-PCQRPLTLLTLMNFQCSAY-LL-

TDSSRRNTVPRWLSWMVVPPRDSVLQLLIISSHWAVKSELIPEYRKLI-ITMEL-RVLY-IMGA-
LKQMRMCSPLQLIS-SFYCLKTGKRCHISRNWRN-LEFQLSMFTYG

>T1026
MAQWACVSAANLSCQATIVNTQKQRNSPGSMPFLSKAVNLWLRAVDFQAHKLFIEGPGMVFAP-
RLFALIIQDQTLTVLLIS-
KLRTSLPLSEPLTRPTKPLKVVIAGAGLAGLATAKYLADAGHQPILLEARDVLGGKDGGLGIIELSAFDQTLLSV
GGGVCVGMGDVLSDRWQHGKIVMGTGMKQACIYSERMMECNSVTNLGMRFLVVSSLHYSPSSVWFKVQTVPGKFK
VAVPNILGVRTPGFPASVFIVIFCPRWIYIIYTLLNETGFGAYPNIQNLFGELGINDRLQWKEHSMIFAMPNKPG
EFSRFDFLEVLPAPINGKYYSHLLKIESSLFTNAENSFCSWEGIWAILKNNEMLTWPEKIKFAIGLLPAILGGQA
YVEAQDGLSVKDWMRKQGIPDRVTTEVFIAMSKALNFINPDELSMQCILIALNRFLQEKHGSKMAFLDGSPPERL
CAPIVDHIQSLGGEVRTNSRIQKIDLNNDGTVKSFVLNNGSVIEADAYVFATPVDILKLLLPENWKEMPYFKKLE
KLVGVPVINVHIW-

>T1027
MAQWACVSAANLSCQATIVNTQKQRNSPGCLFFQRQ-IYGSEL-IFKPTSCL-KAQEWCLPLEGCLR-LSKTRP-
QYC-
FLRSCVLLFHFPSLSRPTKPLKVVIAGAGLAGLATAKYLADAGHQPILLEARDVLGGKDGGLGIIELSAFDQTLL
SVGGGVCVGMGDVLSDRWQHGKIVMGTGMKQACIYSERMMECNSVTNLGMRFLVVSSLHYSPSSVWFKVQTVPGK
FKVAVPNILGVRTPGFPASVFIVIFCPRWIYIIYTLLNETGFGAYPNIQNLFGELGINDRLQWKEHSMIFAMPNK
PGEFSRFDFLEVLPAPINGKYYSHLLKIESSLFTNAENSFCSWEGIWAILKNNEMLTWPEKIKFAIGLLPAILGG
QAYVEAQDGLSVKDWMRKQGIPDRVTTEVFIAMSKALNFINPDELSMQCILIALNRFLQEKHGSKMAFLDGSPPE
RLCAPIVDHIQSLGGEVRTNSRIQKIDLNNDGTVKSFVLNNGSVIEADAYVFATPVDILKLLLPENWKEMPYFKK
LEKLVGVPVINVHIW-

>T1028
MAQWACVSAANLSCQATIVNTQKQRNSPGCLFFQRQ-IYGSEL-IFKPTSCL-KAQEWCLPLEGCLR-LSKTRP-
QYC-FLRSCVLLFHFPSLSLVQPSVKSCDCWCRFGWSGNCKIFGGCGSSTYTTRSERCFRRKGWWVGNY-VECF-
SDTLICWWRGVCWDGGCVV-QVAAWKDSDGDWYETGLHIF-EDDGV-QCNKSGNEIPSS--
LALFSFQCLVQSSDCARKI-GSCT-HLRGSNPRISSICFHCHLLSKVDLHYLHIA--
NWFWGISKYSESVWRAWY-RSVAVEGTFYDICNAKQARGVQSV-FPGSSASTHKW-ILQSPLED-K-
FVYKCRKFFLFLGRNMGHIEEQ-DADLARENQVCNWTTASNPWWAGLC-SPRWLERKRLDEETGHT-
SSNYRGVYSHVKGP-LY-P--TFNAVHIDCFEPIPPGETRFQDGFLGW-SPRETLCSNC-SYPVIGR-SPN-
FPNTEN-SK-RWNCEEFCTK-WERD-SRCVCVRHSS-YLKASIA-KLERDAIFQEIGEISWSSSYQCSHMV

>T1029
MAQWACVSAANLSCQATIVNTQKQRNSPGRCLFFQRQ-IYGSEL-IFKPTSCL-KAQEWCLPLEGCLR-
LSKTRP-QYC-FLRSCVLLFHFPSLSLVQPSR-KL-LLVQVWLVWQLQNIWRMRVINLYY-KREMF-
AERMVGWELLS-VLLIRHSYLLVAGCVLGWGMCCLTGGSMER--WGLV-NRPAYILRG-WSVTV-QIWE-DS--
LVACIILLPVFGSKFRLCQENLR-LYLTS-
GFEPQDFQHLFSLSSSVQGGSTLFTHCLMKLVLGHIQIFRICLESLVLTIGCSGRNIL-

YLQCQTSQGSSVGLISWKFCQHP-MVNITVTS-RLKVVCLQMQKILSVPGKEYGPY-RTMRC-
LGQRKSSLQLDYCQQSLVGRLMLKPKMA-A-KTG-GNRAYLIE-LQRCL-PCQRPLTLLTLMNFQCSAY-LL-
TDSSRRNTVPRWLSWMVVPPRDSVLQLLIISSHWAVKSELIPEYRKLI-ITMEL-RVLY-IMGA-
LKQMRMCSPLQLIS-SFYCLKTGKRCHISRNWRN-LEFQLSMFTYG

>T1030
MAQWACVSAANLSCQATIVNTQKQRNSPGCLFFQRQ-IYGSEL-IFKPTSCL-KAQEWCLPLEGCLR-LSKTRP-
QYC-FLRSCVLLFHFPSLSLVQPSR-KL-LLVQVWLVWQLQNIWRMRVINLYY-KREMF-AERMVGWELLS-
VLLIRHSYLLVAGCVLGWGMCCLTGGSMER--WGLV-NRPAYILRG-WSVTV-QIWE-DS--
LVACIILLPVFGSKFRLCQENLR-LYLTS-
GFEPQDFQHLFSLSSSVQGGSTLFTHCLMKLVLGHIQIFRICLESLVLTIGCSGRNIL-
YLQCQTSQGSSVGLISWKFCQHP-MVNITVTS-RLKVVCLQMQKILSVPGKEYGPY-RTMRC-
LGQRKSSLQLDYCQQSLVGRLMLKPKMA-A-KTG-GNRAYLIE-LQRCL-PCQRPLTLLTLMNFQCSAY-LL-
TDSSRRNTVPRWLSWMVVPPRDSVLQLLIISSHWAVKSELIPEYRKLI-ITMEL-RVLY-IMGA-
LKQMRMCSPLQLIS-SFYCLKTGKRCHISRNWRN-LEFQLSMFTYG

>T1031
MAQWACVSAANLSCQATIVNTQKQRNSPGSMPFLSKAVNLWLRAVDFQAHKLFIEGPGMVFAP-
RLFALIIQDQTLTVLLIS-KLRTSLPLSEPLVQPSR-KL-LLVQVWLVWQLQNIWRMRVINLYY-KREMF-
AERMVGWELLS-VLLIRHSYLLVAGCVLGWGMCCLTGGSMER--WGLV-NRPAYILRG-WSVTV-QIWE-DS--
LVACIILLPVFGSKFRLCQENLR-LYLTS-
GFEPQDFQHLFSLSSSVQGGSTLFTHCLMKLVLGHIQIFRICLESLVLTIGCSGRNIL-
YLQCQTSQGSSVGLISWKFCQHP-MVNITVTS-RLKVVCLQMQKILSVPGKEYGPY-RTMRC-
LGQRKSSLQLDYCQQSLVGRLMLKPKMA-A-KTG-GNRAYLIE-LQRCL-PCQRPLTLLTLMNFQCSAY-LL-
TDSSRRNTVPRWLSWMVVPPRDSVLQLLIISSHWAVKSELIPEYRKLI-ITMEL-RVLY-IMGA-
LKQMRMCSPLQLIS-SFYCLKTGKRCHISRNWRN-LEFQLSMFTYG

>T1032
MAQWACVSAANLSCQATIVNTQKQRNSPGCLFFQRQ-IYGSEL-IFKPTSCL-KAQEWCLPLEGCLR-LSKTRP-
QYC-
FLRSCVLLFHFPSLSLVQPSALKVVIAGAGLAGLATAKYLADAGHQPILLEARDVLGGKDGGLGIIELSAFDQTL
LSVGGGVCVGMGDVLSDRWQHGKIVMGTGMKQACIYSERMMECNSVTNLGMRFLVVSSLHYSPSSVWFKVQTVPG
KFKVAVPNILGVRTPGFPASVFIVIFCPRWIYIIYTLLNETGFGAYPNIQNLFGELGINDRLQWKEHSMIFAMPN
KPGEFSRFDFLEVLPAPINGKYYSHLLKIESSLFTNAENSFCSWEGIWAILKNNEMLTWPEKIKFAIGLLPAILG
GQAYVEAQDGLSVKDWMRKQGIPDRVTTEVFIAMSKALNFINPDELSMQCILIALNRFLQEKHGSKMAFLDGSPP
ERLCAPIVDHIQSLGGEVRTNSRIQKIDLNNDGTVKSFVLNNGSVIEADAYVFATPVDILKLLLPENWKEMPYFK
KLEKLVGVPVINVHIW-

>T1033
MAQWACVSAANLSCQATIVNTQKQRNSPGSMPFLSKAVNLWLRAVDFQAHKLFIEGPGMVFAP-
RLFALIIQDQTLTVLLIS-

KLRTSLPLSEPLTRPTKPLKVVIAGAGLAGLATAKYLADAGHQPILLEARDVLGGKDGGLGIIELSAFDQTLLSV
GGGVCVGMGDVLSDRWQHGKIVMGTGMKQACIYSERMMECNSVTNLGMRFLVVSSLHYSPSSVWFKVQTVPGKFK
VAVPNILGVRTPGFPASVFIVIFCPRWIYIIYTLLNETGFGAYPNIQNLFGELGINDRLQWKEHSMIFAMPNKPG
EFSRFDFLEVLPAPINGKYYSHLLKIESSLFTNAENSFCSWEGIWAILKNNEMLTWPEKIKFAIGLLPAILGGQA
YVEAQDGLSVKDWMRKQGIPDRVTTEVFIAMSKALNFINPDELSMQCILIALNRFLQEKHGSKMAFLDGSPPERL
CAPIVDHIQSLGGEVRTNSRIQKIDLNNDGTVKSFVLNNGSVIEADAYVFATPVDILKLLLPENWKEMPYFKKLE
KLVGVPVINVHIW-

>T1034
MAQWACVSAANLSCQATIVNTQKQRNSPGCLFFQRQ-IYGSEL-IFKPTSCL-KAQEWCLPLEGCLR-LSKTRP-
QYC-FLRSCVLLFHFPSLSLVRPSR-KL-LLVQVWLVWQLQNIWRMRVINLYY-KREMF-AERMVGWELLS-
VLLIRHSYLLVAGCVLGWGMCCLTGGSMER--WGLV-NRPAYILRG-WSVTV-QIWE-DS--
LVACIILLPVFGSKFRLCQENLR-LYLTS-
GFEPQDFQHLFSLSSSVQGGSTLFTHCLMKLVLGHIQIFRICLESLVLTIGCSGRNIL-
YLQCQTSQGSSVGLISWKFCQHP-MVNITVTS-RLKVVCLQMQKILSVPGKEYGPY-RTMRC-
LGQRKSSLQLDYCQQSLVGRLMLKPKMA-A-KTG-GNRAYLIE-LQRCL-PCQRPLTLLTLMNFQCSAY-LL-
TDSSRRNTVPRWLSWMVVPPRDSVLQLLIISSHWAVKSELIPEYRKLI-ITMEL-RVLY-IMGA-
LKQMRMCSPLQLIS-SFYCLKTGKRCHISRNWRN-LEFQLSMFTYG

>T1035
MAQWACVSAANLSCQATIVNTQKQRNSPGSMPFLSKAVNLWLRAVDFQAHKLFIEGPGMVFAP-
RLFALIIQDQTLTVLLIS-KLRTSLPLSEPLLVQPTR-KL-LLVQVWLVWQLQNIWRMRVINLYY-KREMF-
AERMVGWELLS-VLLIRHSYLLVAGCVLGWGMCCLTGGSMER--WGLV-NRPAYILRG-WSVTV-QIWE-DS--
LVACIILLPVFGSKFRLCQENLR-LYLTS-
GFEPQDFQHLFSLSSSVQGGSTLFTHCLMKLVLGHIQIFRICLESLVLTIGCSGRNIL-
YLQCQTSQGSSVGLISWKFCQHP-MVNITVTS-RLKVVCLQMQKILSVPGKEYGPY-RTMRC-
LGQRKSSLQLDYCQQSLVGRLMLKPKMA-A-KTG-GNRAYLIE-LQRCL-PCQRPLTLLTLMNFQCSAY-LL-
TDSSRRNTVPRWLSWMVVPPRDSVLQLLIISSHWAVKSELIPEYRKLI-ITMEL-RVLY-IMGA-
LKQMRMCSPLQLIS-SFYCLKTGKRCHISRNWRN-LEFQLSMFTYG

>T1036
MAQWACVSAANLSCQATIVNTQKQRNSPGCLFFR-KL-LLVQVWLVWQLQNIWRMRVINLYY-KREMF-
AERMVGWELLS-VLLIRHSYLLVAGCVLGWGMCCLTGGSMER--WGLV-NRPAYILRG-WSVTV-QIWE-DS--
LVACIILLPVFGSKFRLCQENLR-LYLTS-
GFEPQDFQHLFSLSSSVQGGSTLFTHCLMKLVLGHIQIFRICLESLVLTIGCSGRNIL-
YLQCQTSQGSSVGLISWKFCQHP-MVNITVTS-RLKVVCLQMQKILSVPGKEYGPY-RTMRC-
LGQRKSSLQLDYCQQSLVGRLMLKPKMA-A-KTG-GNRAYLIE-LQRCL-PCQRPLTLLTLMNFQCSAY-LL-
TDSSRRNTVPRWLSWMVVPPRDSVLQLLIISSHWAVKSELIPEYRKLI-ITMEL-RVLY-IMGA-
LKQMRMCSPLQLIS-SFYCLKTGKRCHISRNWRN-LEFQLSMFTYG

>T1037

MAQWACVSAANLSCQATIVNTQKQRNSRGCLFFQRQ-IYGSEL-IFKPTSCL-KAQEWCLPLEGCLR-LSKTRP-
QYC-FLRSCVLLFHFPSLSLVQPSR-KL-LLVQVWLVWQLQNIWRMRVINLYY-KREMF-AERMVGWELLS-
VLLIRHSYLLVAGCVLGWGMCCLTGGSMER--WGLV-NRPAYILRG-WSVTV-QIWE-DS--
LVACIILLPVFGSKFRLCQENLR-LYLTS-
GFEPQDFQHLFSLSSSVQGGSTLFTHCLMKLVLGHIQIFRICLESLVLTIGCSGRNIL-
YLQCQTSQGSSVGLISWKFCQHP-MVNITVTS-RLKVVCLQMQKILSVPGKEYGPY-RTMRC-
LGQRKSSLQLDYCQQSLVGRLMLKPKMA-A-KTG-GNRAYLIE-LQRCL-PCQRPLTLLTLMNFQCSAY-LL-
TDSSRRNTVPRWLSWMVVPPRDSVLQLLIISSHWAVKSELIPEYRKLI-ITMEL-RVLY-IMGA-
LKQMRMCSPLQLIS-SFYCLKTGKRCHISRNWRN-LEFQLSMFTYG

>T1038
MAQWACVSAANLSCQATIVNTQKQRNSPGCLFFQRQ-IYGSEL-IFKPTSCL-KAQEWCLPLEGCLR-LSKTRP-
QYC-FLRSCVLLFHFPSHSLLQPTR-KL-LLVQVWLVWQLQNIWRMRVINLYY-KREMF-AERMVGWELLS-
VLLIRHSYLLVAGCVLGWGMCCLTGGSMER--WGLV-NRPAYILRG-WSVTV-QIWE-DS--
LVACIILLPVFGSKFRLCQENLR-LYLTS-
GFEPQDFQHLFSLSSSVQGGSTLFTHCLMKLVLGHIQIFRICLESLVLTIGCSGRNIL-
YLQCQTSQGSSVGLISWKFCQHP-MVNITVTS-RLKVVCLQMQKILSVPGKEYGPY-RTMRC-
LGQRKSSLQLDYCQQSLVGRLMLKPKMA-A-KTG-GNRAYLIE-LQRCL-PCQRPLTLLTLMNFQCSAY-LL-
TDSSRRNTVPRWLSWMVVPPRDSVLQLLIISSHWAVKSELIPEYRKLI-ITMEL-RVLY-IMGA-
LKQMRMCSPLQLIS-SFYCLKTGKRCHISRNWRN-LEFQLSMFTYG

>T1039
MAQWACVSAANLSCQATIVNTQKQRNSPGCLFFQRQ-IYGSEL-IFKPTSCL-KAQEWCLPLEGCLR-LSKTRP-
QYC-FLRSCVLLFHFPSLPLVQPSR-KL-LLVQVWLVWQLQNIWRMRVINLYY-KREMF-AERMVGWELLS-
VLLIRHSYLLVAGCVLGWGMCCLTGGSMER--WGLV-NRPAYILRG-WSVTV-QIWE-DS--
LVACIILLPVFGSKFRLCQENLR-LYLTS-
GFEPQDFQHLFSLSSSVQGGSTLFTHCLMKLVLGHIQIFRICLESLVLTIGCSGRNIL-
YLQCQTSQGSSVGLISWKFCQHP-MVNITVTS-RLKVVCLQMQKILSVPGKEYGPY-RTMRC-
LGQRKSSLQLDYCQQSLVGRLMLKPKMA-A-KTG-GNRAYLIE-LQRCL-PCQRPLTLLTLMNFQCSAY-LL-
TDSSRRNTVPRWLSWMVVPPRDSVLQLLIISSHWAVKSELIPEYRKLI-ITMEL-RVLY-IMGA-
LKQMRMCSPLQLIS-SFYCLKTGKRCHISRNWRN-LEFQLSMFTYG
