# Supplementary material for: New Strategies to Overcome Present CRISPR/Cas9 Limitations in Apple and Pear: Efficient Dechimerization and Base Editing
Source: Int J Mol Sci. 2020 Dec 30;22(1):319. doi: 10.3390/ijms22010319 (PMC7795782; doi:10.3390/ijms22010319)
Supplement: Supplementary file 1 [file ijms-22-00319-s001.zip › supplementary/File S2.pdf]

Supplementary material

File S2: pDenCas9_PmCDA1_UGI vector coding sequence developed in this study

The SpnCas9 sequence is in blue, the two NLS sequences in purple, the PmCDA1 sequence in green, the UGI sequence in red, the linker sequences in black and the stop codon in grey. All the coding sequence is optimized for expression in dicot species.

**ATGGATAAGAAGTACTCTATCGGACTCGCTATCGGAACTAACTCTGTGGGATGGGCTGTGATCACCGATGAG
TACAAGGTGCCATCTAAGAAGTTCAAGGTTCTCGGAAACACCGATAGGCACTCTATCAAGAAAAACCTTATC
GGTGCTCTCCTCTTCGATTCTGGTGAAACTGCTGAGGCTACCAGACTCAAGAGAACCGCTAGAAGAAGGTAC
ACCAGAAGAAAGAACAGGATCTGCTACCTCCAAGAGATCTTCTCTAACGAGATGGCTAAAGTGGATGATTC
ATTCTTCCACAGGCTCGAAGAGTCATTCCTCGTGGAAGAAGATAAGAAGCACGAGAGGCACCCTATCTTCGG
AAACATCGTTGATGAGGTGGCATACCACGAGAAGTACCCTACTATCTACCACCTCAGAAAGAAGCTCGTTGA
TTCTACTGATAAGGCTGATCTCAGGCTCATCTACCTCGCTCTCGCTCACATGATCAAGTTCAGAGGACACTTCC
TCATCGAGGGTGATCTCAACCCTGATAACTCTGATGTGGATAAGTTGTTCATCCAGCTCGTGCAGACCTACAA
CCAGCTTTTCGAAGAGAACCCTATCAACGCTTCAGGTGTGGATGCTAAGGCTATCCTCTCTGCTAGGCTCTCT
AAGTCAAGAAGGCTTGAGAACCTCATTGCTCAGCTCCCTGGTGAGAAGAAGAACGGACTTTTCGGAAACTT
GATCGCTCTCTCTCTCGGACTCACCCCTAACTTCAAGTCTAACTTCGATCTCGCTGAGGATGCAAAGCTCCAGC
TCTCAAAGGATACCTACGATGATGATCTCGATAACCTCCTCGCTCAGATCGGAGATCAGTACGCTGATTTGTT
CCTCGCTGCTAAGAACCTCTCTGATGCTATCCTCCTCAGTGATATCCTCAGAGTGAACACCGAGATCACCAAG
GCTCCACTCTCAGCTTCTATGATCAAGAGATACGATGAGCACCACCAGGATCTCACACTTCTCAAGGCTCTTG
TTAGACAGCAGCTCCCAGAGAAGTACAAAGAGATTTTCTTCGATCAGTCTAAGAACGGATACGCTGGTTACA
TCGATGGTGGTGCATCTCAAGAAGAGTTCTACAAGTTCATCAAGCCTATCCTCGAGAAGATGGATGGAACCG
AGGAACTCCTCGTGAAGCTCAATAGAGAGGATCTTCTCAGAAAGCAGAGGACCTTCGATAACGGATCTATCC
CTCATCAGATCCACCTCGGAGAGTTGCACGCTATCCTTAGAAGGCAAGAGGATTTCTACCCATTCCTCAAGGA
TAACAGGGAAAAGATTGAGAAGATTCTCACCTTCAGAATCCCTTACTACGTGGGACCTCTCGCTAGAGGAAA
CTCAAGATTCGCTTGGATGACCAGAAAGTCTGAGGAAACCATCACCCCTTGGAACTTCGAAGAGGTGGTGG
ATAAGGGTGCTAGTGCTCAGTCTTTCATCGAGAGGATGACCAACTTCGATAAGAACCTTCCAAACGAGAAG
GTGCTCCCTAAGCACTCTTTGCTCTACGAGTACTTCACCGTGTACAACGAGTTGACCAAGGTTAAGTACGTGA
CCGAGGGAATGAGGAAGCCTGCTTTTTTGTCAGGTGAGCAAAAGAAGGCTATCGTTGATCTCTTGTTCAAGA
CCAACAGAAAGGTGACCGTGAAGCAGCTCAAAGAGGATTACTTCAAGAAAATCGAGTGCTTCGATTCAGTT
GAGATTTCTGGTGTTGAGGATAGGTTCAACGCATCTCTCGGAACCTACCACGATCTCCTCAAGATCATTAAG
GATAAGGATTTCTTGGATAACGAGGAAAACGAGGATATCTTGGAGGATATCGTTCTTACCCTCACCCTCTTT
GAAGATAGAGAGATGATTGAAGAAAGGCTCAAGACCTACGCTCATCTCTTCGATGATAAGGTGATGAAGCA
GTTGAAGAGAAGAAGATACACTGGTTGGGGAAGGCTCTCAAGAAAGCTCATTAACGGAATCAGGGATAAG
CAGTCTGGAAAGACAATCCTTGATTTCCTCAAGTCTGATGGATTCGCTAACAGAAACTTCATGCAGCTCATCC
ACGATGATTCTCTCACCTTTAAAGAGGATATCCAGAAGGCTCAGGTTTCAGGACAGGGTGATAGTCTCCATG
AGCATATCGCTAACCTCGCTGGATCTCCTGCAATCAAGAAGGGAATCCTCCAGACTGTGAAGGTTGTGGATG
AGTTGGTGAAGGTGATGGGAAGGCATAAGCCTGAGAACATCGTGATCGAAATGGCTAGAGAGAACCAGAC
CACTCAGAAGGGACAGAAGAACTCTAGGGAAAGGATGAAGAGGATCGAGGAAGGTATCAAAGAGCTTGG
ATCTCAGATCCTCAAAGAGCACCCTGTTGAGAACACTCAGCTCCAGAATGAGAAGCTCTACCTCTACTACCTC
CAGAACGGAAGGGATATGTATGTGGATCAAGAGTTGGATATCAACAGGCTCTCTGATTACGATGTTGATCA
TATCGTGCCACAGTCATTCTTGAAGGATGATTCTATCGATAACAAGGTGCTCACCAGGTCTGATAAGAACAG
GGGTAAGAGTGATAACGTGCCAAGTGAAGAGGTTGTGAAGAAAATGAAGAACTATTGGAGGCAGCTCCTC**

AACGCTAAGCTCATCACTCAGAGAAAGTTCGATAACTTGACTAAGGCTGAGAGGGGAGGACTCTCTGAATT
GGATAAGGCAGGATTCATCAAGAGGCAGCTTGTGGAAACCAGGCAGATCACTAAGCACGTTGCACAGATCC
TCGATTCTAGGATGAACACCAAGTACGATGAGAACGATAAGTTGATCAGGGAAGTGAAGGTTATCACCCTC
AAGTCAAAGCTCGTGTCTGATTTCAGAAAGGATTTCCAATTCTACAAGGTGAGGGAAATCAACAACTACCAC
CACGCTCACGATGCTTACCTTAACGCTGTTGTTGGAACCGCTCTCATCAAGAAGTATCCTAAGCTCGAGTCAG
AGTTCGTGTACGGTGATTACAAGGTGTACGATGTGAGGAAGATGATCGCTAAGTCTGAGCAAGAGATCGGA
AAGGCTACCGCTAAGTATTTCTTCTACTCTAACATCATGAATTTCTTCAAGACCGAGATTACCCTCGCTAACGG
TGAGATCAGAAAGAGGCCACTCATCGAGACAAACGGTGAAACAGGTGAGATCGTGTGGGATAAGGGAAG
GGATTTCGCTACCGTTAGAAAGGTGCTCTCTATGCCACAGGTGAACATCGTTAAGAAAACCGAGGTGCAGAC
CGGTGGATTCTCTAAAGAGTCTATCCTCCCTAAGAGGAACTCTGATAAGCTCATTGCTAGGAAGAAGGATTG
GGACCCTAAGAAATACGGTGGTTTCGATTCTCCTACCGTGGCTTACTCTGTTCTCGTTGTGGCTAAGGTTGAG
AAGGGAAAGAGTAAGAAGCTCAAGTCTGTTAAGGAACTTCTCGGAATCACTATCATGGAAAGGTCATCTTT
CGAGAAGAACCCAATCGATTTCCTCGAGGCTAAGGGATACAAAGAGGTTAAGAAGGATCTCATCATCAAGC
TCCCAAAGTACTCACTCTTCGAACTCGAGAACGGTAGAAAGAGGATGCTCGCTTCTGCTGGTGAGCTTCAAA
AGGGAAACGAGCTTGCTCTCCCATCTAAGTACGTTAACTTTCTTTACCTCGCTTCTCACTACGAGAAGTTGAA
GGGATCTCCAGAAGATAACGAGCAGAAGCAACTTTTCGTTGAGCAGCACAAGCACTACTTGGATGAGATCA
TCGAGCAGATCTCTGAGTTCTCTAAAAGGGTGATCCTCGCTGATGCAAACCTCGATAAGGTGTTGTCTGCTTA
CAACAAGCACAGAGATAAGCCTATCAGGGAACAGGCAGAGAACATCATCCATCTCTTCACCCTTACCAACCT
CGGTGCTCCTGCTGCTTTCAAGTACTTCGATACAACCATCGATAGGAAGAGATACACCTCTACCAAAGAAGT
GCTCGATGCTACCCTCATCCATCAGTCTATCACTGGACTCTACGAGACTAGGATCGATCTCTCACAGCTCGGT
GGTGATTCAAGGGCTGATCCTAAGAAGAAGAGGAAGGTTGAATTCGGAGGAGGGCCAGGAGCAGAGTAT
GTTAGAGCGTTGTTTGATTTTAACGGTAACGATGAAGAGGATTTACCCTTTAAGAAAGGCGACATTCTCAGG
ATTAGGGATAAACCTGAAGAGCAATGGTGGAATGCTGAGGATAGTGAAGGCAAACGAGGAATGATTTTAG
TGCCGTATGTGGAGAAATATTCGGGTGACTACAAAGATCATGATGGTGATTACAAAGACCATGACATCGAC
TACAAGGATGATGATGATAAGTCAGGGATGACAGATGCTGAATATGTCAGAATCCACGAAAAGTTGGACAT
TTACACGTTTAAGAAGCAGTTCTTCAACAACAAGAAATCTGTTTCGCATAGGTGCTATGTGCTTTTCGAACTA
AAACGTCGTGGAGAAAGACGGGCTTGCTTTTGGGGTTACGCGGTTAACAAACCACAATCAGGTACTGAACG
AGGAATACACGCTGAAATCTTTTCTATCCGAAAGGTTGAGGAATATCTACGTGACAATCCTGGACAGTTCAC
TATCAATTGGTATTCTAGCTGGTCACCATGTGCAGATTGTGCTGAGAAGATTCTCGAATGGTACAATCAAGA
GCTTAGAGGCAATGGACATACATTGAAAATATGGGCATGCAAGCTCTACTACGAAAAGAATGCCAGAAACC
AAATTGGGCTTTGGAACTTGAGGGATAATGGAGTTGGGCTTAATGTCATGGTTTCTGAGCACTATCAATGTT
GTCGGAAGATCTTCATACAAAGTTCCCATAACCAGTTGAATGAGAACAGATGGTTAGAGAAAACCCTTAAA
AGAGCCGAGAAGAGAAGATCCGAACTGAGCATTATGATACAGGTCAAAATTCTGCATACCACTAAGAGTCC
AGCTGTAGGTCCTAAGAAGAAACGTAAAGTAGGGCCCATGACAAACCTATCAGACATCATTGAAAAAGAAA
CAGGTAAGCAACTCGTCATCCAGGAAAGTATCTTAATGCTCCCTGAAGAAGTCGAGGAGGTGATAGGCAAT
AAGCCTGAAAGTGACATACTTGTCCACACTGCCTATGATGAGTCTACAGACGAGAACGTCATGCTACTTACTT
CAGACGCTCCAGAATACAAACCCTGGGCTCTTGTGATCCAAGATAGTAACGGAGAGAATAAAATAAAAATG
CTGTGA
