# Supplementary material for: New Strategies to Overcome Present CRISPR/Cas9 Limitations in Apple and Pear: Efficient Dechimerization and Base Editing
Source: Int J Mol Sci. 2020 Dec 30;22(1):319. doi: 10.3390/ijms22010319 (PMC7795782; doi:10.3390/ijms22010319)
Supplement: Supplementary file 1 [file ijms-22-00319-s001.zip › supplementary/File S3.pdf]

Supplementary material

File S3

ALS base editing backbone sequences

Bold: promoter, red: target sequence, blue: guide RNA scaffold, italic: termination sequence, lowercase: Gateway attB sites, other: restriction sites, SalI site underlined.

SgRNA ALS

ggggacaagtttgtacaaaaaagcaggcttcatccctattgtcccatta**GAGCTCGAATTCAAAGTAGAACTAGA**
ATGTTTAAATAAAAAGGTTGCTAGTATTCTCTTAACATTAATTAAGCATACGTCCAACTTGTTTATCTTTTAACC
CAAAAACGTATCCCCAATTAACTCACACAGGTTTTTCTGTATAAATTTTAGAGATAGTAGTTTCGGCTCCCAAAT
CTATTGAAAACTAAAGGAACAAAAAACAGAAGAGAGAAGAGGAGCGAGAGCGCTCTTAGCTGTTAAAACGAAACA
TCTCATTTCTTGTCCCACATCGACCGTTTCCAGATTACTAAAGCTGCTTATATGCCTAAACAATGACCAACTGTT
CAAGTA**CCC**CGGAGAATGATGTTTTAGAGCTAGAAATAGCAAGTTAAAATAAGGCTAGTCCGTTATCAACTTGAA
AAAGTGGCACCGAGTCGGTGC*TTTTTTT*GAGCTCGTCgacccagctttcttgtacaaagtggtcccc

PDS base editing backbone sequences

Bold: promoter, red: target sequence, blue: guide RNA scaffold, italic: termination sequence, lowercase: Gateway attB sites, other: restriction sites, XhoI site underlined.

SgRNA MdPDS

ggggacaagtttgtacaaaaaagcaggcttc**GAGCTCCTCGAGTTTTGGATTACCATTCTTAAAATTTGAAGCTG**
TGAATTTGTGTCAACCTATACTTGTACAACATATGACATGCCATATTGTTTCTGTAAGTGGAGTTGTCAACCTGC
CCGTCTAGCTCAGTTGGTAGAGCGCAAGGCTCTTAACCTTGTGGTCGTGGGTTCGAGCCCCACGGTGGGCGCTGC
TTTTTATTTTTAACTTTTTTTAATCGACAAATACGCTGCGTTTTACAAAAAGTGGTGGAGGAGGTGTCCCACATC
GAGCAAACGCAGTGGTATTAATTGCTTTATATTCAATTAGACTGCAAAAAGTGTTGATCAGACACTCTTATCTGT
GTTTTAGAGCTAGAAATAGCAAGTTAAAATAAGGCTAGTCCGTTATCAACTTGAAAAAGTGGCACCGAGTCGGTG
C*TTTTTTT*GAGCTCGAATTCtagggataacagggtaatgacccagctttcttgtacaaagtggtcccc

SgRNA PcPDS

ggggacaagtttgtacaaaaaagcaggcttc**GAGCTCCTCGAGTTTTGGATTACCATTCTTAAAATTTGAAGCTG**
TGAATTTGTGTCAACCTATACTTGTACAACATATGACATGCCATATTGTTTCTGTAAGTGGAGTTGTCAACCTGC
CCGTCTAGCTCAGTTGGTAGAGCGCAAGGCTCTTAACCTTGTGGTCGTGGGTTCGAGCCCCACGGTGGGCGCTGC
TTTTTATTTTTAACTTTTTTTAATCGACAAATACGCTGCGTTTTACAAAAAGTGGTGGAGGAGGTGTCCCACATC
GAGCAAACGCAGTGGTATTAATTGCTTTATATTCAATTAGACTGCAAAAAGTGTTGTTGATCATATCCAGTCATT
GTTTTAGAGCTAGAAATAGCAAGTTAAAATAAGGCTAGTCCGTTATCAACTTGAAAAAGTGGCACCGAGTCGGTG
C*TTTTTTT*GAGCTCGAATTCtagggataacagggtaatgacccagctttcttgtacaaagtggtcccc
