# Supplementary material for: New Strategies to Overcome Present CRISPR/Cas9 Limitations in Apple and Pear: Efficient Dechimerization and Base Editing
Source: Int J Mol Sci. 2020 Dec 30;22(1):319. doi: 10.3390/ijms22010319 (PMC7795782; doi:10.3390/ijms22010319)
Supplement: Supplementary file 1 [file ijms-22-00319-s001.zip › supplementary/Table S1.pdf]

Supplementary material

Table S1. Primer information concerning dechimerization and base editing assays

Sequence	Accessions/references	Forward primer 5'-3'	Reverse primer 5'-3'	Annealing temperature
**Cloning control and/or transgenic status control**				
pUC/M13 pGEM-T Easy plasmid	Thermo Fisher (Fermentas)	CGCCAGGGTTTTCCCAGTCACGAC	CAGGAAACAGCTATGAC	55°C
nCas9	Tan *et al.,* 2020	ATCAACGCGTATGGATAA	GAAGTACTCTATCGGACTC	56°C
Elongation Factor Malus domestica	AJ223969	CTCTTGGTGTCAGGCAAATG	TCAAGGTTGGTGGACCTCTC	60°C
23S ribosomal RNA Agrobacterium tumefaciens (UF/B1R)	CP014260.1 gene locus_tag="AWN88_17620" 1310643..1313449	GTAAGAAGCGAACGCAGGGAACT	GACAATGACTGTTCTACGCGTAA	66°C
nptII plasmid pK7WG2D	Karimi *et al.,* 2002	ATCGGGAGCGGCGATACCGTA	GAGGCTATTCGGCTATGACTG	60°C
cloning box in pDONR207 plasmid	Thermo Fisher (Invitrogen)	TCGCGTTAACGCTAGCATGGATCTC	GTAACATCAGAGATTTTGAGACAC	60°C
**Target sequences cloning and analysis**				
MdPDS (dechimerization)	MD04G0021400* GDDH13	GTCAAGCAAGACAGAATGGA	GACCTCTAAATGGCTGCTACTG	60°C
MdALS/ PcALS (CBE)	MD06G1028700* GDDH13 /PCP000109.1_v1.0 Bartlet in GDR	GCGTGTGCATTGCAACCT	CCTAACAACCCTAGGAATGTCCTC	63°C
PcPDS (CBE)	PCP034470.1_v1.0 Bartlet in GDR	GGAGAAACACGGTTCCAAGATG	GAGTGGCAAACACATACGCATC	63°C
MdPDS (CBE)	MD04G0021400* GDDH13	AGTGGGCTTGTGTCTCCG	CCGCCTAAAACATCTCTCGC	58°C

* available at https://iris.angers.inra.fr/gddh13
